# The effect of telemedicine employing telemonitoring instruments on readmissions of patients with heart failure and/or COPD: a systematic review

**DOI:** 10.3389/fdgth.2024.1441334

**Published:** 2024-09-25

**Authors:** Georgios M. Stergiopoulos, Anissa N. Elayadi, Edward S. Chen, Panagis Galiatsatos

**Affiliations:** ^1^ Department of Molecular Medicine, Mayo Clinic, Rochester, MN, United States; ^2^ Research and Exploratory Development, Johns Hopkins University Applied Physics Laboratory, Laurel, MD, United States; ^3^ Division of Pulmonary and Critical Care Medicine, The Johns Hopkins School of Medicine, Baltimore, MD, United States

**Keywords:** telemedicine, telemonitoring, readmission(s), heart failure, ADHF -acute decompensated heart failure, COPD, AECOPD -acute exacerbation of chronic obstructive pulmonary disease

## Abstract

**Background:**

Hospital readmissions pose a challenge for modern healthcare systems. Our aim was to assess the efficacy of telemedicine incorporating telemonitoring of patients’ vital signs in decreasing readmissions with a focus on a specific patient population particularly prone to rehospitalization: patients with heart failure (HF) and/or chronic obstructive pulmonary disease (COPD) through a comparative effectiveness systematic review.

**Methods:**

Three major electronic databases, including PubMed, Scopus, and ProQuest's ABI/INFORM, were searched for English-language articles published between 2012 and 2023. The studies included in the review employed telemedicine incorporating telemonitoring technologies and quantified the effect on hospital readmissions in the HF and/or COPD populations.

**Results:**

Thirty scientific articles referencing twenty-nine clinical studies were identified (total of 4,326 patients) and were assessed for risk of bias using the RoB2 (nine moderate risk, six serious risk) and ROBINS-I tools (two moderate risk, two serious risk), and the Newcastle-Ottawa Scale (three good-quality, four fair-quality, two poor-quality). Regarding the primary outcome of our study which was readmissions: the readmission-related outcome most studied was all-cause readmissions followed by HF and acute exacerbation of COPD readmissions. Fourteen studies suggested that telemedicine using telemonitoring decreases the readmission-related burden, while most of the remaining studies suggested that it had a neutral effect on hospital readmissions. Examination of prospective studies focusing on all-cause readmission resulted in the observation of a clearer association in the reduction of all-cause readmissions in patients with COPD compared to patients with HF (100% vs. 8%).

**Conclusions:**

This systematic review suggests that current telemedicine interventions employing telemonitoring instruments can decrease the readmission rates of patients with COPD, but most likely do not impact the readmission-related burden of the HF population. Implementation of novel telemonitoring technologies and conduct of more high-quality studies as well as studies of populations with ≥2 chronic disease are necessary to draw definitive conclusions.

**Systematic Review Registration:**

This study is registered at the International Platform of Registered Systematic Review and Meta-analysis Protocols (INPLASY), identifier (INPLASY202460097).

## Background

Telemedicine is a multidisciplinary, interactive, and continuously evolving tool first introduced into the medical practice during the past century (1). Recently, the COVID-19 outbreak has promoted and accelerated the incorporation of telemedicine into healthcare systems all over the world (2). Telemedicine is often described as the use of communication networks for the delivery of healthcare services and medical education from one geographic location to another (1). The instruments used by telemedicine often involve communication and/or surveillance technologies (3), with the latter being referred to as telemonitoring (4). Indicatively, some communication tools employed are videoconferences, telephone calls, text messages, and mobile app alerts, while home telemonitoring usually involves vital sign monitoring with medical devices like pulse oximeters, blood pressure (BP) cuffs, spirometers, thermometers, and electrocardiographs. These devices can be directly connected via Bluetooth or Wi-Fi to a transmitting device, or they can be regular devices requiring the patient to manually transfer their data to a digital interface accessible by the healthcare team (5, 6).

Identifying a patient population to gain an equitable advantage with telemedicine is vital. One population would, in theory, be those experiencing recent hospitalization. As hospital readmissions pose great economic, social, and psychological issues for patients and their families (7, 8) and still remain one of the main preventable financial strains in modern healthcare systems (9), investigating the effect of telemedicine on hospital readmissions is essential for understanding its potential advantages in healthcare. In the present study we focused on two chronic conditions that contribute significantly to hospital readmissions: heart failure (HF) and chronic obstructive pulmonary disease (COPD) (10, 11).

HF is a clinical syndrome in which symptoms occur due to functional or structural impairment of ventricular filling or ejection of blood (12). It is one of the most prevalent diseases with more than 38 million people suffering worldwide (13) and is characterized by recurrent hospitalizations due to decompensation of the cardiac function (14) accounting for approximately 1%–2% of all hospital admissions in Europe and North America (15). Acute decompensated HF (ADHF) is often referred to as new or worsening signs and symptoms of HF that often lead to emergency department (ED) visits or hospitalizations and is usually associated with systemic congestion (16). Studies suggest that about a quarter of patients with HF are readmitted within 30 days upon discharge (17). Moreover, patients usually fail to understand the initial signs of decompensation and ultimately reach the ED once the ADHF has progressed and serious dyspnea has developed (18, 19). The signs of early ADHF could be easily identified with BP, heart rate (HR), oxygen saturation (Ox Sat), and weight monitoring and addressed by adjustment of the patient's medication regimen, preventing a hospital readmission. Daily monitoring of patients in the outpatient setting is feasible solely with the assistance of telemedicine which is why it has been considered to have a promising role in patient management following an ADHF episode (20).

COPD is characterized by persistent airflow limitation due to airway and/or alveolar abnormalities with the most common risk factor being tobacco smoking (21). Acute exacerbation of COPD (AECOPD), as defined by the Global Initiative for Chronic Obstructive Lung Disease (GOLD) is an acute event characterized by a worsening of the patient's respiratory symptoms that is beyond normal day-to-day variations leading to a change in medication (21). Patients with AECOPD often require hospitalization (22) which accounts for about 70% of total COPD-related medical costs (23). Apart from the great financial burden, AECOPD might have potentially severe consequences for patients, such as a decline in pulmonary function (24) and increase in mortality (25). The rate of readmissions following an AECOPD is particularly high, notably, a study conducted in the US found a 64% readmission rate after a discharge for an AECOPD in Medicare beneficiaries (26). The high rate of readmissions propagating the financial, psychological, and medical load alongside the patients’ difficulty to properly recognizing the early symptoms of deterioration (27) mandate the need for the development of an effective system to recognize the onset of an exacerbation timely.

Furthermore, HF and COPD frequently coexist as comorbidities, and their concurrent presence is associated with increased readmissions and mortality (28, 29), thus we aimed to assess the presence of current literature on the effectiveness of telemedicine utilizing telemonitoring instruments in reducing hospital readmissions for patients with both HF and COPD.

Many studies have investigated telemedicine's effectiveness in areas such as mortality, healthcare utilization (e.g., readmission, ED visits), patient satisfaction, and quality of life (QoL) in the HF and COPD population (30–35). These studies employ various telemedicine technologies, either alone or in combination, including communication devices, mobile health applications and remote vital sign monitoring (30–32, 36). Reports systematically assessing clinical trials in these populations suggest that telemedicine can be an effective strategy for reducing readmission rates in these patients (30, 31, 33, 34, 36) and also highlight the need for further research focused on achieving reliable and reproducible clinical outcomes (37).

Given recent advances in the field of remote monitoring, we believe that the future of telemedicine will be closely intertwined with telemonitoring. Therefore, this systematic review aims to assess the current literature on the effectiveness of telemedicine employing remote monitoring technologies in reducing hospital readmissions for patients with HF and/or COPD. Additionally, our study is the first of its kind to provide comparative data regarding the efficacy of telemedicine in these two common groups of patients, as well as evaluate the presence of evidence for patients with comorbid cardiopulmonary disease.

## Methods

### Data sources

We conducted a comparative effectiveness systematic review of journal articles published between 2012 and 2023 following the Preferred Reporting Items for Systematic Reviews and Meta-Analyses (PRISMA) guidelines. Data collection was completed on December 31, 2023. We searched for English-language articles identified through PubMed, Scopus, and ProQuest's ABI (Abstracted Business Information)/INFORM.

### Search strategy

Our search strategy on the databases above included the following: hospital readmission(s) OR patient readmission(s) OR readmission or readmissions OR re-admissions AND telemedicine OR smartphone(s) OR telehealth OR digital health OR eHealth OR health application(s) OR mHealth OR health app(s) OR mobile application(s) OR mobile app(s) OR portable electronic app OR smartphone app(s) OR smartphone. When applicable, we limited the search to adults (in PubMed) and excluded wire feeds, blogs, newspapers, magazines, dissertations, and working papers from the results (in ABI/INFORM). The exact research strings used for each database can be found in the Supplemental Materials.

### Eligibility criteria

Inclusion and exclusion criteria followed the Participants-Intervention-Comparison-Outcome (PICO) framework (38): 1. Participants were defined as adults (≥18 years old) who had been previously hospitalized and diagnosed with HF and/or COPD. Studies including patients <18 years old or patients who were not hospitalized before the initiation of the intervention were excluded. 2. Interventions consisted of telemedicine incorporating remote vital sign monitoring. Studies not using any type of telemonitoring (e.g., the only interventions were follow-up calls, texts, and video visits) were excluded. 3. The comparison was HF and/or COPD patients receiving usual care. Studies comparing telemedicine interventions with inpatient hospitalization, or in-person rehabilitation were excluded. 4. The outcomes included any readmission-related outcome. The primary outcome of our study were readmission-related outcomes which encompassed incidence or rate of all-cause readmissions, HF-readmissions, AECOPD-readmissions, time to first readmission, days alive spent in the hospital, composite endpoints of readmission and mortality, etc. Secondary outcomes in our systematic review were either associated with the healthcare burden (e.g., ED visits, outpatient visits, and total healthcare costs) or linked to patient benefits (e.g., mortality, QoL assessed through standardized questionnaires, medication adherence, and medication reconciliation). When none of the outcomes was related to readmissions/re-hospitalizations, the studies were excluded.

### Study identification

GS and PG finalized the research criteria, and GS reviewed all titles and abstracts identified from the search strategy. Full texts (*n* = 40) were subsequently independently reviewed by GS and PG and 10 additional papers were excluded. Three studies were excluded because patients were not hospitalized before the beginning of the study (*n* = 3), four studies were excluded because the comparison group was hospitalization or in-person rehabilitation (*n* = 4). One study was excluded because the comparison group received a telemedicine intervention as well (*n* = 1). Moreover, another study was excluded because it was an analysis of systems of an ongoing RCT (*n* = 1). Furthermore, two publications (*n* = 2) were excluded because readmissions were not the primary or secondary endpoint of the studies.

### Data extraction

Data extraction from full-text articles (*n* = 30) was done independently by two reviewers (PG and GS) and included: disease of the patient population studied, type of study, type of telemedicine and telemonitoring intervention, participant characteristics, comparison group intervention, number of patients enrolled in each group, country where the study took place, primary and secondary endpoints of the studies, study outcomes related to readmissions, and other relevant study outcomes.

During the process of data extraction for our primary outcome, we encountered variations in the ways different studies quantified the impact on readmissions. This variability prohibited the conduction of a meta-analysis of the data, so our results were summarized narratively. We qualitatively assessed outcomes such as the rate or number of readmissions (both all-cause and disease-specific), the time to first readmission (both all-cause and disease-specific), and the duration of subsequent hospitalizations (both all-cause and disease-specific). Furthermore, studies focusing on the HF population, COPD population, or HF and COPD population were analyzed independently.

### Quality assessment

Risk of bias assessment was done using Cochrane-developed tools such as risk of bias tool for randomized trials (RoB2) for randomized controlled trials (RCTs) (39) and risk of bias in non-randomized studies of interventions (ROBINS-I) for non-RCTs (40). Utilization of the ROBINS-I tool necessitates a proactive identification of potential confounders. Through discussion and literature review, other than disease-specific indicators of readmission risk (e.g., disease severity, frequency and duration of hospitalizations, other comorbidities), we determined that age, sex, socioeconomic status, technology, and health literacy were important domains of potential confounding that could affect the results of telemedicine interventions on readmissions. For cohort studies the Newcastle-Ottawa scale (NOS) for cohort studies was used and for the case-control studies the Newcastle-Ottawa scale for case-control studies was employed. Bias assessment for each study was conducted with respect to the reported readmission-related outcome(s).

The reporting of readmission-related outcomes varied significantly across studies. Even for all-cause readmissions, which were the focus of most studies, there was no standardized approach to reporting outcomes. Various studies presented outcomes in different formats, including absolute numbers or percentage of all-cause readmissions per patient, number of events within specific time frames (e.g., 360 days, 180 days, or 30 days), hazard ratios, odds ratios, readmission rates, or composite endpoints such as all-cause readmissions or death. Consequently, a graphical or statistical assessment of reporting bias was not feasible.

Overall judgement of the certainty/confidence of our results was done independently by two reviewers (PG and GS) and separately for HF and COPD studies using the Grading of Recommendations Assessment, Development and Evaluation (GRADE) criteria for effects which have been summarized narratively (41).

## Results

**Figure 1:**
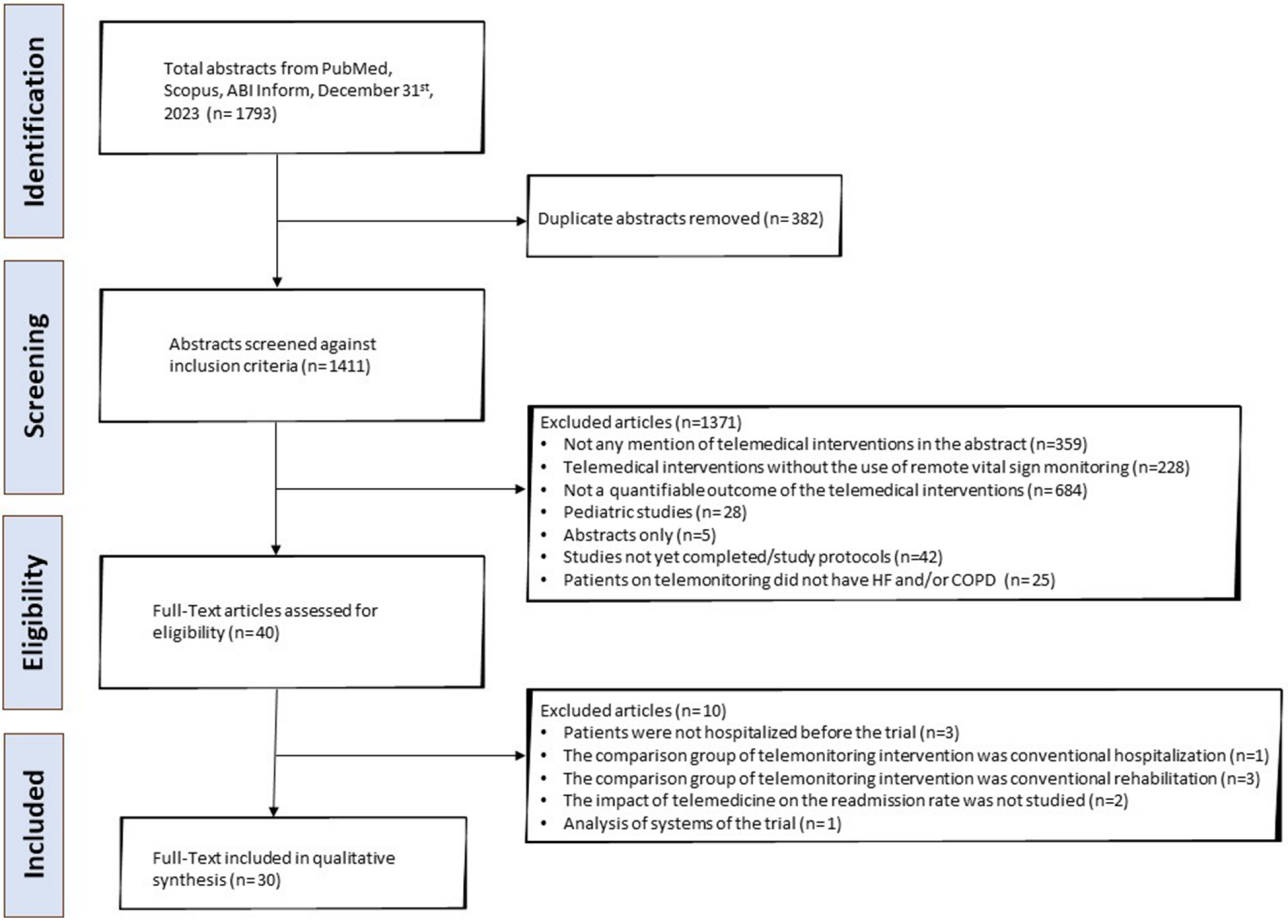
PRISMA flowchart depicting the process of the literature search of the systematic review. PRISMA, Preferred Reporting Items for Systematic Reviews and Meta-Analyses.

**Figure 2:**
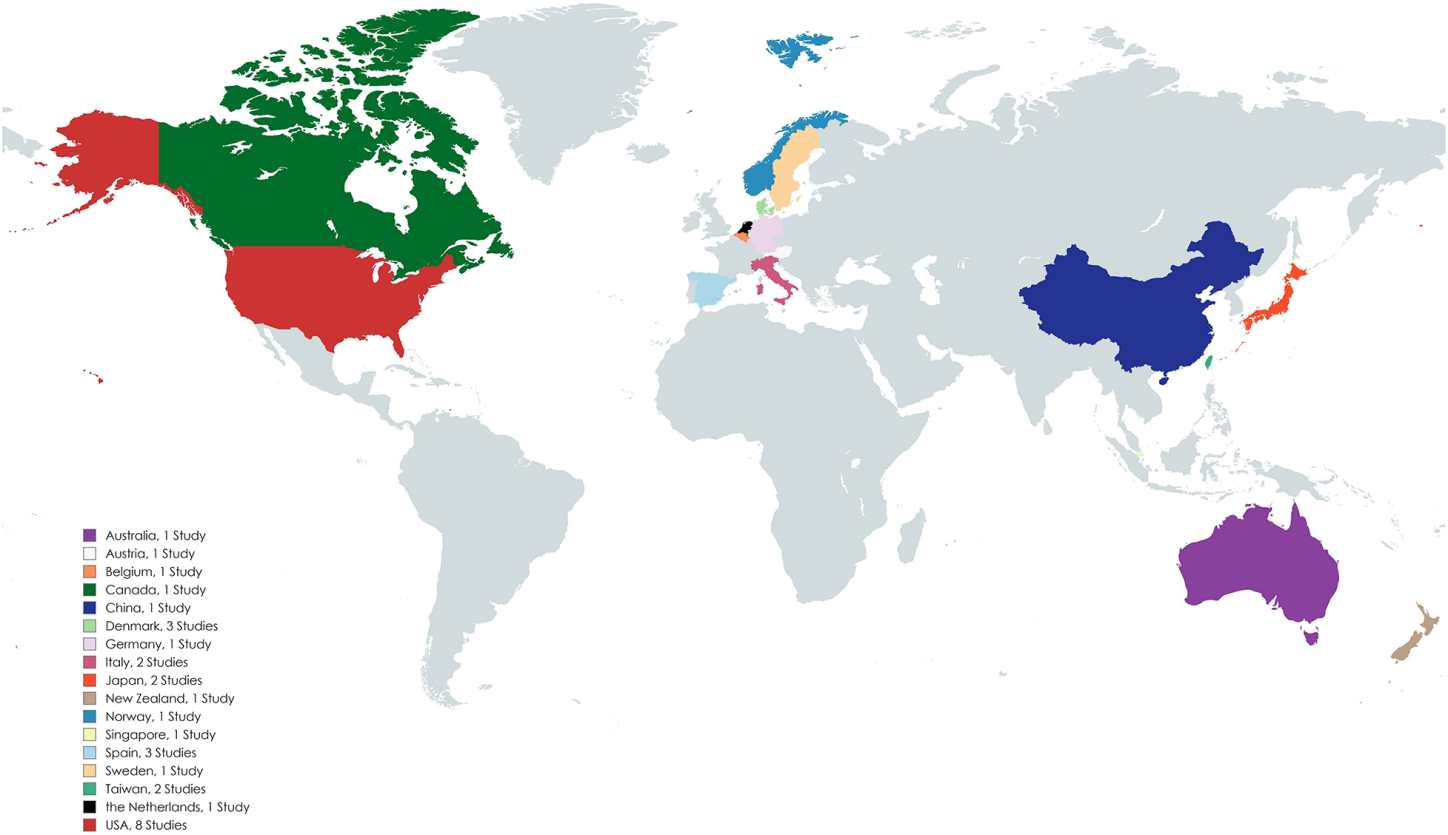
World map representing the countries where the identified studies were conducted.

A total of 1,411 publications were isolated, of which 1,381 were screened out as described in the “Study Identification” section of the “Methods” and shown in the PRISMA flowchart (Figure 1). The data extracted from the remaining articles (*n* = 30) are displayed in Table 1 and refer to twenty-nine different studies (42–71). Two of the articles refer to the same trial (short- and long-term analysis), so their data are presented in the same table row (57, 58). The majority of these studies were conducted in Europe and North America (*n* = 22) (42–45, 47–51, 53, 55–58, 61–63, 65–70) (Figure 2). Most focused on HF (*n* = 17) (42, 46–48, 50–55, 57–59, 62, 65, 68–70) and fewer focused on COPD (*n* = 10) (43–45, 49, 56, 60, 63, 64, 66, 71). Additionally, one study enrolled patients with HF and COPD (*n* = 1) (61) and another one patients with HF or COPD (*n* = 1) (67). The results from the latter study are presented separately for each condition in the table, and they are assessed according to the respective disease in the results section. Most studies were RCTs (*n* = 15) (43, 45, 48, 50, 54, 57–62, 64, 65, 69–71). Some were non-RCT (*n* = 4) (46, 52, 56, 63), cohort studies (*n* = 9) (42, 44, 47, 49, 51, 53, 66–68), two non-specified feasibility studies (*n* = 2) (51, 53), and one was a case-control study (*n* = 1) (55).

**Table 1:** Overview of the results.

Author, Year	Country	Disease	Type of study	Risk of bias (ROB2 or ROBINS-I)/quality (NOS)	Telemedicine intervention	Participant characteristics	Comparison group	No. of patients	Outcomes (Primary or Secondary Endpoint)	Readmission-related outcomes (rate/time to/duration) (Primary or Secondary Endpoint)	Other relevant outcomes	Ref. No.
Parikh et al., 2023	USA	HF	Retrospective Cohort	Good-quality	Telemonitoring (HR, Ox Sat, BP, weight)	Mean age was 75 ± 11 years, 55% were male	Usual care (*n* = 1,985) (matched)	726	30-, 90-, 365-day HF-readmissions 30-, 90-, 365- all-cause mortality (Primary) 30-, 90-, 365-day, all-cause readmissions, 30-, 90-, 365-day diuretic adjustment. (Secondary)	No significant difference in 30-, 90-, 365-day HF-readmissions (adjusted association: 0.95[0.68, 1.33], 0.99 [0.78, 1.25], 1.08 [0.90, 1.29] respectively) (Primary) No significant difference in 30-, 90-, 365-day, all-cause readmissions (adjusted association: 0.82 [0.65, 1.05], 0.92 [0.77, 1.11], 1.02 [0.89, 1.116] respectively) (Secondary)	Significant increase in the 30-, 90-, 365-day diuretic adjustment (adjusted association: 1.84 [1.44, 2.36], 1.76 [1.42, 2.16], 1.54 [1.29, 1.89] respectively) No significant difference in 30-, 90-, 365- all-cause mortality readmissions (adjusted association: 0.60 [0.33, 1.0.5], 0.85 [0.60, 1.20], 1.00 [0.80, 1.25] respectively)	(42)
Zanaboni et al., 2023	Norway, Australia, Denmark	COPD	RCT	Moderate	Telemonitoring (HR, Ox sat) +Tele-rehabilitation (treadmill)	Mean age was 64.9 ± 7.1 years, 57.5% were male, 70% severe-very severe obstruction per GOLD	Usual care (*n* = 40)	40	All-cause readmission or ED visits [composite endpoint, (Primary)], all-cause readmission ED visits, 6MWD, CAT, MRC, GSES, EQ5D, EQ-VAS (Secondary)	Significant decrease in all-cause readmission or ED visits (composite endpoint, IRR: 0.63, *p* = 0.0008) (Primary) Significant decrease in all-cause readmissions (IRR: 0.057, *p* = 0.0002) (Secondary)	Significant decrease in ED visits (IRR: 0.064, *p* = 0.0022), no significant difference in 1-year, 6MWD (mean ± SD: 415 ± 146, *p* = 0.209), CAT (mean ± SD: 18.7 ± 0.089, *p* = 0.209), MRC (mean ± SD: 1.8 ± 1.2, *p* = 0.089), GSES (mean ± SD: 30.5 ± 5.5, *p* = 0.462), EQ5D (mean ± SD: 0.671 ± 0.214, *p* = 0.0.903), EQ-VAS (mean ± SD: 56.3 ± 18.9, *p* = 0.653)	(43)
Naya Prieto et al., 2023	Spain	COPD	Cohort	Poor-quality	Telemonitoring (HR, Ox Sat, Temperature) + symptom questionnaires	Mean age was 75.56 ± 9.57 years, 71.42% were male, 59.1% severe–very severe obstruction per GOLD	Usual care (*n* = 73)	25	AECOPD-readmissions, ED visits, length of hospital stay	No significant difference in AECOPD-readmissions (20% vs. 23, 38%, *p* = 0.734) and length of hospital stay (7.8 vs. 7.2, *p* = 0.789)	No significant difference in ED visits (16% vs. 26%, *p* = 0.307)	(44)
Andersen et al., 2023	Denmark	COPD	RCT	Serious	Telemonitoring (HR, Ox sat, PEF, weight)	Median age was 70 (range 64–76), 38% were male, 17% moderate, 54% severe, 29% very severe obstruction per GOLD	Usual care (*n* = 112)	110	180-day AECOPD-readmission (Primary) 730-day AECOPD-readmissions, 180-day and 730-day days in the hospital, time to first readmission (Secondary)	Significant decrease in the 180-day AECOPD readmissions (IRR: 1.43, *p* = 0.03) (Primary) No significant difference in the 730-day AECOPD readmissions (IRR: 1.04, *p* = 0.644), 180-day or 730-day days in the hospital (IRR: 1.22, *p* = 0.2, 1.08, *p* = 0.285 respectively), or time to first readmission (Haz R: 1.23, CI 0.77–1.99, *p* = 0.4)	–	(45)
Tsai et al., 2022	Taiwan	HF	Non-RCT	Serious	Telemonitoring (HR, Ox Sat, ECG, weight) + tele-rehabilitation (cycle ergometers, stepper, elliptical cross trainers, walking training)	Mean age was 73.3 ± 5.0, 70.7% were male, mean LVEF: 33.5 ± 11.2%, mean 6MWD 250.8 ± 92.6 m	Usual care (*n* = 43)	42	Functional capacity (Δ6MWD) (Primary) LVEF, 30-day and 90-day all-cause readmission rate (Secondary)	No significant difference in 30-day and 90-day all-cause readmission rate [4.3%, and 4.6% decrease compared to usual care respectively, not statistically significant (*p* not provided)] (Secondary)	Significant improvement in functional capacity (Δ6MWD, 51.2% vs. 17.7% *P* < 0.05), ΔLVEF (25.6% relative increase, *p* < 0.05)	(46)
Poelzl et al., 2022	Austria	HF	Retrospective cohort	Good-quality	Telemonitoring (HR, BP, weight)	Mean age was 69.5 ± 11.9 years, 25% were male, mean LVEF 36.8 ± 13.8%, NYHA: II (26.3%), III (72.5%), IV (1.2%)	Usual care (*n* = 257) (retrospectively matched)	251	180-day HF-readmission or all-cause mortality [composite endpoint, (Primary)] 30- and 90-day HF-readmission, HF-readmission rate per 100 person-years, all-cause mortality, *Δ*NYHA class, self-efficacy (ΔEHFScBs-9) (Secondary)	Significant decrease in 180-day HF-readmission or all-cause mortality (Haz R 0.54 *p* < 0.001) [composite endpoint, (Primary)] Significant decrease in 90-day HF-readmission (7.2% vs. 13.9%, *p* = 0.006), significant decrease in the HF-readmission rate per 100 person-years (rate ratio: 0.41 *p* < 0.001) no significant decrease in 30-day HF-readmission (10.5% vs. 23.7%, *p* = 0.185) (Secondary)	Significant decrease in all-cause mortality (Haz R = 0.38, *p* < 0.0001), significant decrease in ΔNYHA class (reduction to baseline 1.9 ± 0.71, *p* < 0.001), significant improvement in self-efficacy (ΔEHFScBs-9, from baseline of 22.4 ± 6.0 to 11.3 ± 2.5, *p* < 0.001)	(47)
Völler et al., 2022	Germany	HF	RCT	Moderate	Telemonitoring (HR, BP, weight, questionnaires) + health coaching (interactive device)	Mean age was 63.0 ± 11.5 years, 88% were male, mean LVEF was 30.4 ± 7.4%, mean 6-MWD was 375 ± 132 m	Usual care (*n* = 319)	302	Days alive neither in hospital nor inpatient care per potential days in study, incremental cost-effectiveness ratio (Primary) All-cause readmissions, all-cause mortality and health related QoL (Secondary)	No significant difference in days alive neither in hospital nor inpatient care per potential days (mean: 341 ± 59 days vs. 346 ± 45 days, *p* = 0.298) (Primary)	No significant difference in cost-effectiveness ratio (probability of cost-effectiveness was <14.4%), significant increase of QoL (by SF-36 [estimated difference physical component 2, *p* = 0.009, mental component 2.7, *p* = 0.004] WHO-5 [estimated difference 1.3 *p* = 0.01] and KCCQ [overall summary, estimated difference 5.5 *p* < 0.001]) at 6 and 12 months in the telemedicine group	(48)
Marcos et al., 2022	Spain	COPD	Retrospective cohort	Good-quality	Telemonitoring (HR, Ox sat, clinical questions)	Mean age was 68.7 ± 9.4 years, 85.8% were male, 3.7% mild, 25.6% moderate, 41.2% severe, 29.4% very severe obstruction per GOLD	Usual care (retrospective propensity score analysis) (*n* = 495)	351	All-cause mortality or AECOPD readmissions [composite endpoint, (Primary)] AECOPD readmissions at 1-, 3-, 6-, and 12-month; time to readmission, time to death and hospital length of stay. (Secondary)	Significant reduction in all-cause mortality or AECOPD readmission after 12 months [35.2% vs. 45.2%, Haz R of 0.71 (95% CI = 0.56–0.91), *p* = 0.007] (Primary) the time to the composite outcome longer (197.8 vs. 142.7 Haz R = 0.54 (95% IC = 0.35–0.84) (Secondary)	–	(49)
Dorsch et al., 2021	USA	HF	RCT	Moderate	Telemonitoring (weight, physical activity) + mobile app	Mean age was 60.2 ± 9.2 years, 63% were male, LVEF 38.8%, NYHA: II (12%), III (66%), IV (22%)	Usual care (*n* = 41)	42	6- and 12-week ΔMLHFQ score (Primary) HF-readmissions and SCHFI questionnaire score (Secondary)	No significant difference in HF-readmission (Haz R 0.89, 95% CI 0.39–2.02, *p* = 0.78) (Secondary)	Significant reduction in ΔMLHFQ score at 6 weeks (mean 37.5 vs. 48.2, *p* = .04) but not at 12 weeks (44.2 vs. 45.9, *p* = .78), no significant difference in the ΔSCHFI at 6 or 12 weeks (change from baseline: 186.1 vs. 187.8, *p* = 0.40, 196.9 vs. 206.1, *p* = 0.24 respectively)	(50)
Ho et al.*,* 2021	Canada	HF	Feasibility (recruiting prospectively)	Fair- quality	Telemonitoring (HR, BP, weight)	Median age was 75 years (range was 44–93), 51% were male.	Self-90-days before intervention (*n* = 70)	70	90-day ED visits or all-cause mortality [composite endpoint, (Primary)] 90-day all-cause readmissions, hospitalization cost reduction, QoL (ΔVR-12, ΔKCCS), self-efficacy (ΔEHFScBs-9) Secondary	No significant difference in 90-day all-cause readmissions (−87% pre- vs. post-telemedicine implementation, *p* not provided) (Secondary)	No significant difference in 90-day ED visits or all-cause mortality (no effect measure or *p* value provided), significant reduction in hospitalization costs (71%, *p* < 0.001), significant improvement in QoL (in the physical and mental component of ΔVR-12) (19% increase in both, *p* = 0.02 and *p* < 0.01 respectively), in ΔKCCS (101% increase, *p* < 0.001, no significant improvement in self-efficacy (ΔEHFScBs-9, 5.84% increase, *p* = 0.22)	(51)
Leng-Chow et al., 2020	Singapore	HF	Non-RCT	Moderate	Telemonitoring (HR, BP, weight, questionnaires) + health coaching (interactive device)	Median age was 57.9 ± 12.3 years, 60.7% were male, LVEF 32.8 ± 16.9%, NYHA: I (20.8%), II (56.5%), III + IV (22.6%)	Usual care + structured telephone support (*n* = 55)	150	180-day and 360-day all-cause, all-cause HF bed days, all-cause 365-day mortality, and cost	No significant differences in 180-day and 360-day all-cause -readmission rates (Haz R = 0.62, CI: 0.28–1, *p* = 0.05, Haz R = 0,73 (CI:.47–1.13, *p* = 0.16 respectively) Significant reduction of all-cause 180-day bed days (5 vs. 9.8 days, *p* = 0.03), HF bed days (1.2 vs. 6 days *p* < 0.01), one-year HF bed days (2.2 vs. 6.6, *p* = 0.02),	Significant reduction in all-cause 365-day mortality (Haz R = 0.32, *p* = 0.02) significantly higher estimated mean maintenance and confidence scores at 1 year (71.4 vs. 61.5, *p* < 0.001, 65.4 vs. 59, *p* < 0.01) reduction in one-year total cost of care (−2,774.4 Singapore dollars *p* = 0.07) in the telemedicine group, no significant differences in knowledge level (11.6 vs. 10.9, *p* = 0.06)	(52)
Park et al.*,* 2019	USA	HF	Feasibility	Poor-quality	Telemonitoring (BP, weight)	Median age was 62 years, 67% were male	National average	58	30-day all-cause readmissions (Primary)	No significant difference in 30-day all-cause readmissions (10% vs. 23%, *p* = 0.06) (Primary)	–	(53)
Mizukawa et al.*,* 2019	Japan	HF	RCT	Serious	Telemonitoring (HR, BP, weight)	Mean age was 70.5 ± 13.3 years, 50% were male, LVEF 42.2 ± 16.7%, NYHA III-IV: 55%	Usual care (*n* = 19)	20	QoL (MLWHFQ) (Primary) Self-efficacy scale (CDSES), self-care (EHFScBs-9, HF-readmissions, all-cause readmissions, all-cause mortality, HF-readmission, or all-cause mortality [composite endpoint, (Secondary)]	Significant decrease in HF-readmissions (20% vs. 57.9%, *p* = 0.048), no significant decrease in all-cause readmissions (60% vs. 68.4%, *p* = 0.902) No significant difference HF-readmission or all-cause mortality (composite endpoint, 30% vs. 63.2%, *p* = 0.068)	Significant increase in the QoL at 18 and 24 months (*p* = 0.014, *p* = 0.016, respectively), significant improvement in 6 and 18 months in self-efficacy (ΔCDSES *p* = 0.001, *p* = 0.020, respectively) and at 6, 12, and 18 months, in self-care (ΔEHFScBs-9, *p* = 0.005, *p* = 0.021, *p* = 0.022, respectively) No significant difference in all-cause mortality (15 vs. 15.8, *p* = 0.859)	(54)
Srivastava et al., 2019	USA	HF	Retrospective case-control	Fair-quality	Telemonitoring (HR, BP, weight)	Median age 74 (range 46–98), 98% were male	Usual care group (*n* = 870) and self-1-year before telemonitoring initiation (*n* = 197)	197	All-cause/HF- readmissions, total hospital days per patient, length of stay per admission, urgent care, ED and primary care visits	Significant reduction in all-cause readmissions (1.1 vs. 1.6 in self-1-year before telemonitoring, *p* < 0.05) but not with usual care group. Significant reduction in the length of hospital stay in the telemedicine group (5.7 vs. 9.5, *p* < 0.01) Significant reduction in total hospital days per patient (2.4 vs. 4.1, *p* < 0.0001 and 3.8, *p* < 0.001) self-1-year before telemonitoring and control respectively, admission rate (1.1 vs. 1.6, *p* < 0.05) and length of stay (5.7 vs. 9.5, *p* < 0.01) with self-1-year before telemonitoring but not in comparison with the usual care group (5.7 ± 11.3 vs. 9.0 ± 14.9, *p* < 0.01). No significant differences in HF-readmissions	No significant difference during telemedical monitoring compared to the prior year in the number of urgent care and ED visits (mean: 2.5 vs. 2.3, *p* = 0.51), or primary care visits (mean: 2.6 vs. 3, *p* = 0.08)	(55)
Bhatt et al., 2019	USA	COPD	Non-RCT	Serious	Telemonitoring (HR, BP, Ox Sat,) in relation to tele-rehabilitation (smartphone foot pedaler (± treadmills, exercise bikes)	Median age 64.5%, 38.7% were male, enrollment irrespective of disease severity, mean FEV1% of predicted 45.4 ± 18.1%	Usual care (matched by readmission risk/not randomized/contemporaneous) (*n* = 160)	80	30-day all-cause readmissions (Primary) 30-day AECOPD readmissions (Secondary)	Significant reduction in 30-day all-cause readmission rate (6.2% vs. 18.1%, *p* = 0.013) (Primary), significant reduction in 30-day AECOPD readmission rate (3.8% vs. 11.9% *p* = 0.04) (Secondary)	–	(56)
Dendale et al., 2012 Frederix et al., 2019	Belgium	HF	RCT	Moderate	Telemonitoring (HR, BP, weight)	Mean age 76 ± 10 years, 65% were male, mean LVEF 35 ± 15%	Usual care (*n* = 80)	80	All-cause mortality (Primary) All-cause readmissions/patient. Days lost due to all-cause readmissions, HF-readmissions, death, or dialysis, related costs comparison, and number of hospitalizations (Secondary)	Significant reduction of total days lost due to hospitalization (13 vs. 30, *p* = 0.02) during the intervention, the reduction was maintained in the long-term analysis (7.28 vs. 11.81, *p* = 0.04) HF-readmissions per patient showed a trend (0.24 vs. 0.42, *p* = 0.06) hospitalizations/patient, (0.51 vs. 0.70, *p* = 0.06) in favor of telemedicine, no significant differences in all-cause readmissions/patient (0.80 vs. 0.82, *p* = 0.93) (Secondary)	Significant reduction in all-cause mortality (5% vs. 17.5%, *p* = 0.01), for the 6-month duration of the intervention, significant reduction in all-cause mortality not validated in the long-term analysis, no significant difference in cost (mean 2,557 vs. 2,643, *p* = 0.90)	(57, 58)
Kotooka et al., 2018	Japan	HF	RCT	Moderate	Telemonitoring (HR, BP, weight, body composition)	Mean age was 67.1 ± 12.8 years, 51% were male, only NYHA: II-III (70/20)	Usual care (*n* = 91)	90	HF-readmissions and all-cause mortality [Composite endpoint, (Primary)] all-cause and cardiovascular- readmissions, mortality from cardiovascular causes, cost of medical care, cardiac biomarkers, MMSE, GSES, MLWHF PHQ-9 scores, and adherence to medication (Secondary)	No significant difference in HF-readmissions (Haz R = 1.007, CI:0.534–1.897 *p* = 0.983) (Primary), no significant reduction in all-cause and cardiovascular readmissions (Haz R = 0.795, CI: 0.479–1.320, *p* = 0.376, Haz R = 0.595, CI:0.171–2.074, *p* = 0.415, respectively) (Secondary)	No significant difference in all-cause mortality (Haz R = 0.809 CI: 0.354–1.847, *p* = 0.614) and the mortality from cardiovascular causes (Haz R = 0.524 CI: 0.176–1.557, *p* = 0.245), cost of medical. Care, cardiac biomarkers (e.g., LVEF *p* = 0.922) MMSE (*p* = 0.568), GSES (*p* = 0.842), MLWHF (*p* = 0.943), PHQ-9 scores (*p* = 0.498), or adherence to medication (no *p* provided).	(59)
Broadbent et al., 2018	New Zealand	COPD	RCT	Serious	Telemonitoring (HR, Ox Sat, FEV1,) + CCQ + reminders for medication and tele-rehabilitation (socially assistive robot)	Mean age was 70.57 ± 10.34 years, 37% were male, 37% were classified as severe and 50% as very severe obstruction per GOLD Additionally, recruited patients had poor social support, lived in remote location and leaved house <4 times per week.	Usual Care (*n* = 30)	30	Respiratory-related readmissions and cost-effectiveness analysis (Primary) Medication adherence and QoL (Secondary)	No significant difference in respiratory-related readmissions (absolute number 15 vs. 15, *p* > 0.99) (Primary)	Significant increase in medication adherence (48.5% vs. 29.5%, *p* = 0.03), no significant difference in CCQ score (functional *p* = 0.11, symptoms *p* = 0.33, mental *p* = 0.5, total *p* = 0.36) (QoL), cost of care (mean difference 1,152 New Zealand $, CI: −760 to 3,356, *p* = 0.32).	(60)
Bernocchi et al., 2017	Italy	HF + COPD	RCT	Serious	Telemonitoring (HR, Ox Sat, ECG) + tele-rehabilitation + educational interventions + structured phone calls	Mean age was 71 years, 88% were male, with HF-NYHA: II-IV and COPD-GOLD: moderate-very severe obstruction.	Usual care (*n* = 56)	56	Exercise tolerance (6MWT) (Primary) time to all-cause readmission or mortality (Composite endpoint), dyspnea (MRC), physical activity profile (PASE), disability (Barthel) and QoL (MLHFQ, CAT) (Secondary)	Significant increase in the median time to all-cause readmission or mortality [Composite endpoint, (113.4 vs. 104.7 days, *p* = 0.0484)] (Secondary)	Significant improvement in the Δ6MWT from baseline to +60 vs. −15 m, *p* = 0.004, in ΔMRC (−0.17 vs. 0.07, *p* = 0.0500), ΔBarthel (5.4 vs. 1.3, *p* = 0.0006), ΔMLHFQ (−10.5 vs.−0.44, *p* = 0.0007) and ΔCAT (−5.3 vs. −1.6, *p* = 0.00001)	(61)
Ong et al., 2016	USA	HF	RCT	Moderate	Telemonitoring (HR, BP, weight) + health coaching (telephone calls, interactive device)	Median age was 73 years, 53.8% were male, LVEF: 42.7 ± 1.3%, NYHA: I (0.2%), II (23.4%), III (65.6%), IV (10.8%)	Usual care (*n* = 722)	715	180-day all-cause readmissions (Primary) 30-day all-cause readmission 30- and 180-day all-cause mortality 30- and 180-day QoL (Secondary)	No significant difference in 180-day all-cause readmission (Haz R = 1.03 95% CI, 0.88–1.20, *p* = 0.74) (Primary), no significant difference in 30-day all-cause readmission (Haz R = 1.01, 95% CI: 0.80–1.28, *p* = 0.91) (Secondary)	No significant difference in all-cause mortality (Haz R = 0.61, 95% CI: 0.37–1.02, *p* = 0.06), significant increase in 180-day QoL (MLHFQ) (28.5 vs. 32.63, *p* = 0.02)	(62)
Esteban et al., 2016	Spain	COPD	Non-RCT	Moderate	Telemonitoring (HR, RR, Ox Sat, questionnaires) + mobile app + patient education	Mean age was 70.1 ± 7.5 years, 87.2% were male, having COPD with ≥2 AECOPD admission last year or ≥3 within the past 2 years	Usual care (*n* = 78)	119	AECOPD readmission rate, length of stay, 30-day all-cause readmission rate (Primary) ED visits, mortality, QoL, exercise capacity, limitations in daily life (Secondary)	Significant reduction of AECOPD readmissions (OR 0.38, 95% CI 0.27–0.54, *p* < 0.0001) (Primary), 30-day all-cause readmissions (OR 0.46, 95% CI 0.29–0.74, *p* < 0.001) and length of hospital stay, (OR 0.58, 95% CI 0.46–0.73, *p* < 0.0001) (Secondary)	Significant reduction in ED visits (OR 0.56, 95% CI 0.35–0.92, *p* < 0.02)	(63)
Ho et al., 2016	Taiwan	COPD	RCT	Moderate	Telemonitoring (HR, BP, Ox Sat, temperature, weight) + symptom diary	Mean age was 81.4 ± 7.8 years, 81% were male 66% mild-moderate, 34% severe-very severe obstruction per GOLD	Usual care (*n* = 53)	53	Time to first AECOPD readmissions within 6 months (Primary) all-cause readmissions per patient, ED visits (Secondary)	Significant increase of time to first AECOPD readmission within 6 months (*p* = 0.02) (Primary) all-cause readmissions (0.23 vs. 0.68/patient; *p* = 0.002) (Secondary)	Significant reduction in ED visits (0.36 vs. 0.91/patient; *p* = 0.006)	(64)
Kraai et al., 2016	the Netherlands	HF	RCT	Moderate	Telemonitoring (HR, BP, weight, ECG) + ICT-guided- DMS	Mean age was 69 ± 12 years, 72% were male, mean LVEF was 28 ± 9%, NYHA: II (21%), III (51%), IV (18%)	ICT-guided- DMS (*n* = 83)	94	HF-readmission or mortality [Composite endpoint, (Primary)] all-cause readmissions, all-cause mortality, QoL, HF outpatient clinic visits, cost analysis (Secondary)	No significant difference in all-cause readmissions (49% vs. 51%, *p* = 0.78) or HF-readmissions (28% vs. 27% *p* = 0.87) (Secondary)	No significant difference in composite endpoint score, all-cause mortality (mean difference 0.1, 95%CI: −0.67 to 0.82, *p* = 0.39), QoL (−14 vs. −15, 95% CI: −8.7- 7.4, *p* = 0.63), cost analysis, significant reduction of visits to the HF-outpatient clinic (2 vs. 4, *p* = 0.02)	(65)
Dyrvig et al., 2015	Denmark	COPD	Retrospective cohort	Fair-quality	Telemonitoring (HR, Ox Sat, Spirometer) + Teleconsultation with a nurse	Median age was 71.80 years, 39.52% were male	Usual Care (Dataset of 11.303 patients)	210	Readmissions, risk of all-cause mortality during the observation period	Significant increase in all-cause readmission during the RCT (OR = 3.44, 95% CI: 2.59–4.57, *p* < 0.0001) (Especially: female sex and old age)	Significantly lower risk of all-cause mortality during trial (Haz R = 0.65, 95% CI: 0.47–0.88, *p* = 0.006)	(66)
Davis, et al., 2015	USA	HF	Retrospective cohort	Fair-quality	Telemonitoring (HF: weight) +Questionnaires	Mean age was 63 ± 16.6 years, 54.2% were male, patients defined as underserved	Usual care (*n* = 59)	59	30-, 90-, 180-day all-cause readmission, ED visits (Primary)	No significant difference in 30-, 90-, 180-day all-cause readmission for HF (OR = 0.45, 95%CI: 0.15–1.42, *p* = 0.09, OR = 0.72, 95% CI: 0.32- 1.60, *p* = 0.12, OR = 0.70, 95% CI: 0.32- 1.60, *p* = 0.10, respectively	No significant in 30-, 90-, 180-day ED visits (OR = 1.85, 95%CI: 0.51–6.70, *p* = 0.16, OR = 0.91, 95% CI: 0.36- 2.23, *p* = 0.18, OR = 0.61, 95% CI: 0.27- 1.36, *p* = 0.08, respectively)	(67)
	USA	COPD	Retrospective cohort	Fair-quality	Telemonitoring (Ox Sat, HR, weight) + Questionnaires	Mean age was 61 ± 11 years, 37.9% were male, patients defined as underserved	Usual care (*n* = 174)	71	30-, 90-, 180-day all-cause readmission, ED visits (Primary)	Significant decrease in 30-day all-cause readmissions for COPD (OR = 0.41, 95%CI:0.17–1.04, *p* = 0.02), no significant difference for 90-, 180-day all-cause readmission for COPD (OR = 0.86 95% CI: 0.47–1.69, *p* = 0.11, and OR = 0.76, 95% CI: 0.42–1.38, *p* = 0.08 respectively) (Primary)	No significant in 30-, 90-, 180-day ED visits (OR = 0.61, 95%CI: 0.20–1.86, *p* = 0.15, OR = 0.90, 95% CI: 0.44- 1.87, *p* = 0.15, OR = 0.97, 95% CI: 0.51- 1.85, *p* = 0.13, respectively)	
Thomason et al., 2015	USA	HF	Retrospective cohort	Fair-quality	Telemonitoring (HR, BP, Ox Sat, weight)	Median age was 84 years, 40% were male	Usual care (*n* = 1,337)	70	All-cause readmissions	Non-significant decrease in all-cause readmission rate (10% vs. 22%, no *p* value provided, assumed to be non-significant)	-	(68)
Villani et al.*,* 2014	Italy	HF	RCT	Serious	Telemonitoring (as determined by treating cardiologist: HR, BP, weight, ECG)	Mean age was 71 years, 88% were male with at least two of: age > 70 years, > 2 hospitalizations for heart failure in the last 6 months, > 1 co-pathologies (diabetes, COPD, cerebrovascular disease, renal failure)	Usual care (*n* = 40)	40	All-cause readmissions, HF-readmission for >3 days, ΔNYHA class, Δpulmonary artery pressure psychological status (ΔSTAI-6, ΔPHQ-9, ΔPGWBI, ΔMMAS)	Significant decrease in all-cause readmissions (17 vs. 6, *p* < 0.02), significant decrease in HF-readmission for >3 days (23 vs. 12, *p* < 0.02).	Significant decrease in ΔNYHA class (2.1 vs. 2.4, *p* < 0.02), Δpulmonary artery pressure (38 vs. 33, *p* < 0.05), improved psychological status (ΔSTAI-6, ΔPHQ-9, ΔPGWBI, ΔMMAS, *p* < 0.01, *p* < 0.01, *p* < 0.01, 0 < 0.05 respectively)	(69)
Lynga et al., 2012	Sweden	HF	RCT	Moderate	Telemonitoring (weight)	Mean age was 73 ± 10.2 years, 75.9% were male, LVEF <30% (61.4%), 30–39% (20.5%), 40–49% (18.1%), NYHA III (96.4%), IV (3.6%)	Usual care (*n* = 153)	166	HF-readmissions (Primary) all-cause readmissions, all-cause mortality, composite endpoint of all-cause readmissions and all-cause mortality (Secondary)	No significant difference in HF-readmissions (Haz R = 0.90, 95%CI:0.65–1.26, *p* = 0.54) (Primary) no significant difference in all-cause readmissions (Haz R = 0.83, 95% CI: 0.61–1.13, *p* = 0.24) (Secondary)	No significant difference in all-cause mortality (Haz R = 0.57, 95%CI: 0.19–1.73, *p* = 0.32), composite endpoint of all-cause readmissions and all-cause mortality (Haz R = 0.90, 95%CI 0.65–1.26, *p* = 0.54)	(70)
Chau et al., 2012	China	COPD	RCT	Serious	Telemonitoring (HR, RR, Ox Sat)	Mean age was 73.50 ± 6.05, 95.5% were male, with moderate (18.2%), severe (40.9%), very severe (40.9) obstruction per GOLD	Usual care (*n* = 18)	22	AECOPD-readmissions, pulmonary function, ED visits, pulmonary function	No significant difference in AECOPD-readmissions (no *p* value provided)	No significant difference in pulmonary function (FEV1/FVC) (no *p* value provided), ED visits. (no *p* value provided)	(71)

AECOPD, acute exacerbation of COPD; BP, blood pressure; CCQ, Clinical COPD Questionnaire; CDSES, chronic disease self-efficacy scale; COPD, chronic obstructive pulmonary disease; CAT, COPD assessment test; CI, confidence interval; ECG, Electrocardiogram; GSES, general self-efficacy scale; GOLD, global initiative for chronic obstructive lung disease; Haz R, hazard ratio; HF, heart failure; HR, heart rate; EQ-5D, EuroQol 5 Dimensions; EQ-VAS, EuroQol Visual Analog Scale; EHFScBs-9, European Heart Failure Self-Care Behavior Scale 9; FEV1, forced expiratory volume; IRR, incidence risk ratio; ICT-guided-DMS, information and computing technology-guided-disease-management system; KCCQ, Kansas City Cardiomyopathy Questionnaire; LVEF, left ventricular ejection fraction; MMAS, Morisky Medical Adherence Scale; MRC, Medical Research Council; MLHFQ, Minnesota Living With Heart Failure Questionnaire; MMSE, mini mental state examination score, NOS, Newcastle-Ottawa Scale; NYHA, New York Heart Association; OR, odds ratio; Ox sat, Oxygen saturation; PASE, physical activity scale for the elderly; PHQ-9, Patient Health Questionnaire; PEF, peak expiratory flow; PGWBI, perceived general well being index; QoL, quality of life; RCT, randomized controlled trial; RR, respiratory rate; RoB2, risk of bias tool for randomized trials; ROBINS-I, risk of bias in non-randomized studies of interventions; SCHFI, self-care heart failure index; SF-36, Short Form-36; SD, standard deviation; STAI, state-trait anxiety inventory; VR-12, Veterans Rand 12-item Health Survey; WHO-5, World Health Organization Well Being Index; 6MWT, 6-min Walk Test; 6MD, 6-min Walk Distance.

### Types of telemonitoring interventions employed

**Figure 3:**
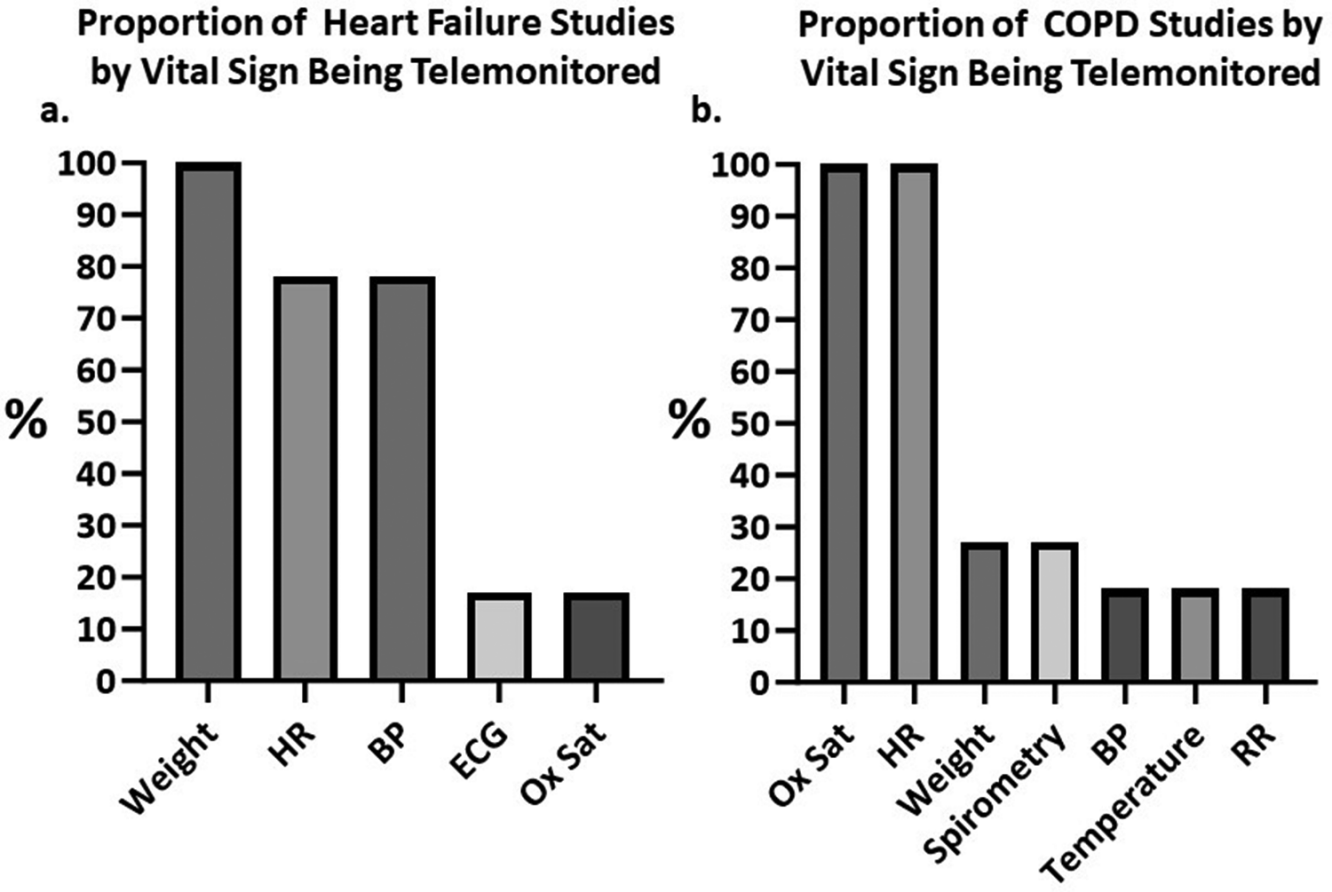
Column charts depicting the percentages of identified studies using remote monitoring for each vital sign. **(A)** Proportion of heart failure (HF) studies telemonitoring for weight, heart rate (HR), blood pressure (BP), electrocardiograph (ECG) and oxygen saturation (Ox Sat). **(B)** Proportion of chronic obstructive pulmonary diseases (COPD) studies telemonitoring for Ox Sat, HR, weight, pulmonary volumes via spirometry, BP, temperature and respiratory rate (RR). BP, blood pressure; COPD, chronic obstructive pulmonary disease; ECG, Electrocardiograph; HF, heart failure; HR, heart rate; Ox Sat, oxygen saturation; RR, respiratory rate.

The studies identified utilized various combinations of telemonitoring modalities. A visual representation of the vital signs monitored in HF and COPD studies is shown in Figure 3. Among the vital signs most frequently monitored were the HR (*n* = 26) (78% of HF studies, 100% of COPD studies) (42–49, 51, 52, 54–69, 71), body weight (*n* = 20) (100% of HF studies, 27% of COPD studies) (42, 45–48, 50–55, 57–59, 62, 64, 65, 67–70), BP (*n* = 15) (78% of HF studies, 18% of COPD studies) (42, 47, 48, 51–59, 62, 64, 65, 68, 69), and Ox Sat (*n* = 14) (17% of HF studies, 100% of COPD studies) (42–46, 49, 56, 60, 61, 63, 64, 66–68, 71). Less commonly, temperature (*n* = 2) (0% of HF studies, 18% of COPD studies) (44, 64), forced expiratory volume (FEV1) (0% of HF studies, 9% of COPD studies) (60), peak expiratory flow (PEF) (*n* = 1) (0% of HF studies, 9% of COPD studies) (45), respiratory rate (RR) monitoring (*n* = 2) (0% of HF studies, 18% of COPD studies) (63, 71), and electrocardiogram (ECG) monitoring (*n* = 4) (17% of HF studies, 0% of COPD studies) (46, 61, 65, 69) were employed. No trend was observed in the achievement of readmission-related outcomes based on the vital sign monitored (Supplementary Figure S1). This is likely due to most studies monitoring for the same vital signs (e.g., almost all HF studies monitored weight, BP, and HR, and all COPD studies monitored Ox Sat and HR). Additionally, there were very few studies employing other modalities (e.g., ECG or Ox Sat for HF, and temperature or RR for COPD) hindering the interpretation of any observed changes.

Furthermore, a subset of studies utilized daily questionnaires and clinical inquiries (*n* = 8) (17% of HF studies, 55% of COPD studies) (44, 48, 49, 52, 60, 63, 64, 67). Other than the variability of the telemonitoring devices used, there were differences in the communication channels used for remote consultation such as telephone calls, messages, smartphone apps or video consultation. Some studies (*n* = 5) combined telemonitoring with telerehabilitation (6% of HF studies, 27% of COPD studies) (43, 46, 56, 60, 61).

### Readmission-related outcomes

Approximately half of the studies (48%) reported a positive impact, to some extent, of telemedicine on hospital readmissions (*n* = 14) (43, 45, 47, 49, 52, 54–57, 61, 63, 64, 67, 69). Conversely, most remaining studies (*n* = 14) (42, 44, 46, 48, 50, 51, 53, 59, 60, 62, 65, 68, 70, 71) did not observe a statistically significant difference between the group undergoing telemonitoring and the control group. Additionally, a low-quality study (*n* = 1) (66) indicated that patients in the telemedicine group had a higher risk of readmission during the telemedicine intervention. Notably, among the higher-quality studies (*n* = 9) (43, 48, 50, 57, 59, 62, 64, 65, 70), only three studies (*n* = 3, 33%) (43, 57, 64) suggested that the telemedicine interventions might decrease readmissions. Two of the latter studies exclusively targeted the COPD population (*n* = 2, 100%) (43, 64), whereas the other study examined the HF population (*n* = 1, 14%) (57). Conversely, the remaining higher-quality studies, all of which investigated the HF population did not identify a similar effect (48, 50, 59, 62, 65, 70).

### Readmission-related outcomes in studies of the heart failure (HF) population

Our results demonstrate that 67% (*n* = 12) (42, 46, 48, 50, 51, 53, 54, 59, 62, 65, 67, 68, 70) of the studies focusing on the HF population (*n* = 18) (42, 46–48, 50–55, 57–59, 62, 65, 67–70) did not report any statistically significant effect on readmissions in the intervention group. The remaining 33% (*n* = 6) (47, 52, 54, 55, 57, 69) found telemedicine interventions to be effective at decreasing either the duration and rate of readmissions or increasing the time to first readmission.

Out of the prospective HF studies (*n* = 13) (46, 48, 50–54, 57, 59, 62, 65, 69) only 31% (*n* = 4) (52, 54, 57, 69) suggested that there might be a readmission-related benefit in the intervention group (decrease in all-cause readmissions, HF-readmission for >3 days, 180-day bed days, or days lost due to hospitalization). Additionally, for all-cause readmissions; eleven out of the twelve prospective studies examining all-cause readmissions as a primary or secondary outcome in the HF population (*n* = 11, 92%) (46, 48, 51–54, 57, 59, 62, 65, 70) concluded that telemedicine interventions had a neutral effect on this endpoint. Considering the reproducible outcomes reported by several large, moderate risk of bias RCTs, along with fewer higher risk of bias studies pointing in the opposite direction, we express moderate confidence in reporting that the telemedicine interventions including telemonitoring are unlikely to significantly impact readmissions in patients with HF (Table 4).

### Readmission-related outcomes in studies of the chronic obstructive pulmonary disease (COPD) population

Our results demonstrate that 64% (*n* = 7) (43, 45, 49, 56, 63, 64, 67) of the studies focusing on the COPD populations (*n* = 11) (43–45, 49, 56, 60, 63, 64, 66, 67, 71) demonstrated a readmission-related benefit. Specifically, 100% of the prospective studies which studied all-cause readmissions (*n* = 4) (43, 56, 63, 64) reported a statistically significant decrease in the intervention group. In relation to the prospective studies assessing AECOPD-readmissions (*n* = 6) (44, 45, 56, 60, 63, 64), 67% (*n* = 4) (45, 56, 63, 64) reported a decrease in the telemedicine group. The two trials (*n* = 2) (44, 60) that did not demonstrate a statistically significant decrease were characterized by small sample sizes and serious risk of bias, evaluating ≤30 patients each. Only one study (*n* = 1, 14%) (66), with serious risk of bias, suggested that people in the telemedicine group were more likely to be readmitted to the hospital. Considering the reproducible outcomes reported by the larger lower risk of bias prospective studies we report with poor confidence that the telemedicine interventions including telemonitoring employed within the last decade could be effective at decreasing the readmission rates in patients with COPD (Table 4).

### Readmission-related outcomes in studies including patients with heart failure (HF) and chronic obstructive pulmonary disease (COPD)

Only one study (*n* = 1) was identified that examined patients with comorbid HF and COPD (61). This study reported a significant increase in the median time to all-cause readmission or death in this patient population. More studies are warranted to determine whether patients with ≥2 chronic disease could derive benefits from telemedicine interventions.

### Other relevant endpoints

Some of the identified studies also reported lower risk of all-cause mortality (*n* = 4; *n* = 3 for HF, *n* = 1 for COPD) (47, 52, 57, 66), improved QoL (*n* = 6; *n* = 5 for HF, *n* = 1 for HF and COPD) (48, 50, 51, 54, 61, 62), reduced ED visits (*n* = 3; all for COPD) (43, 63, 64), decreased cost (*n* = 2; all for HF) (51, 52), improvement in the New York Heart Associations functional class (NYHA, *n* = 2; all for HF) (47, 69), or the left ventricular ejection fractions (LVEF, *n* = 1; all for HF) (46) higher self-efficacy or confidence levels (*n* = 3; all for HF) (47, 52, 54), exercise tolerance (*n* = 2; *n* = 1 for HF, *n* = 1 for HF and COPD) (46, 61), and improved medication adherence and reconciliation (*n* = 1; all for COPD) (60) in the telemedicine groups. However, it is crucial to note the ambiguity in some of these results, as other studies suggested that there was no significant difference in all-cause mortality (*n* = 7; all for HF) (42, 51, 54, 59, 62, 65, 70), ED visits (*n* = 5; *n* = 2 for COPD, *n* = 2 for HF, *n* = 1 for HF or COPD) (44, 51, 55, 67, 71), healthcare costs (*n* = 4; *n* = 3 for HF, *n* = 1 for COPD) (48, 57, 60, 65), QoL (*n* = 2; *n* = 1 for HF, *n* = 1 for COPD) (43, 59, 60), self-efficacy (*n* = 3; *n* = 2 for HF, *n* = 1 for COPD) (43, 50, 51), and medication adherence (*n* = 1; all for HF) (59).

### Risk of bias assessment and certainty of evidence

**Figure 4:**
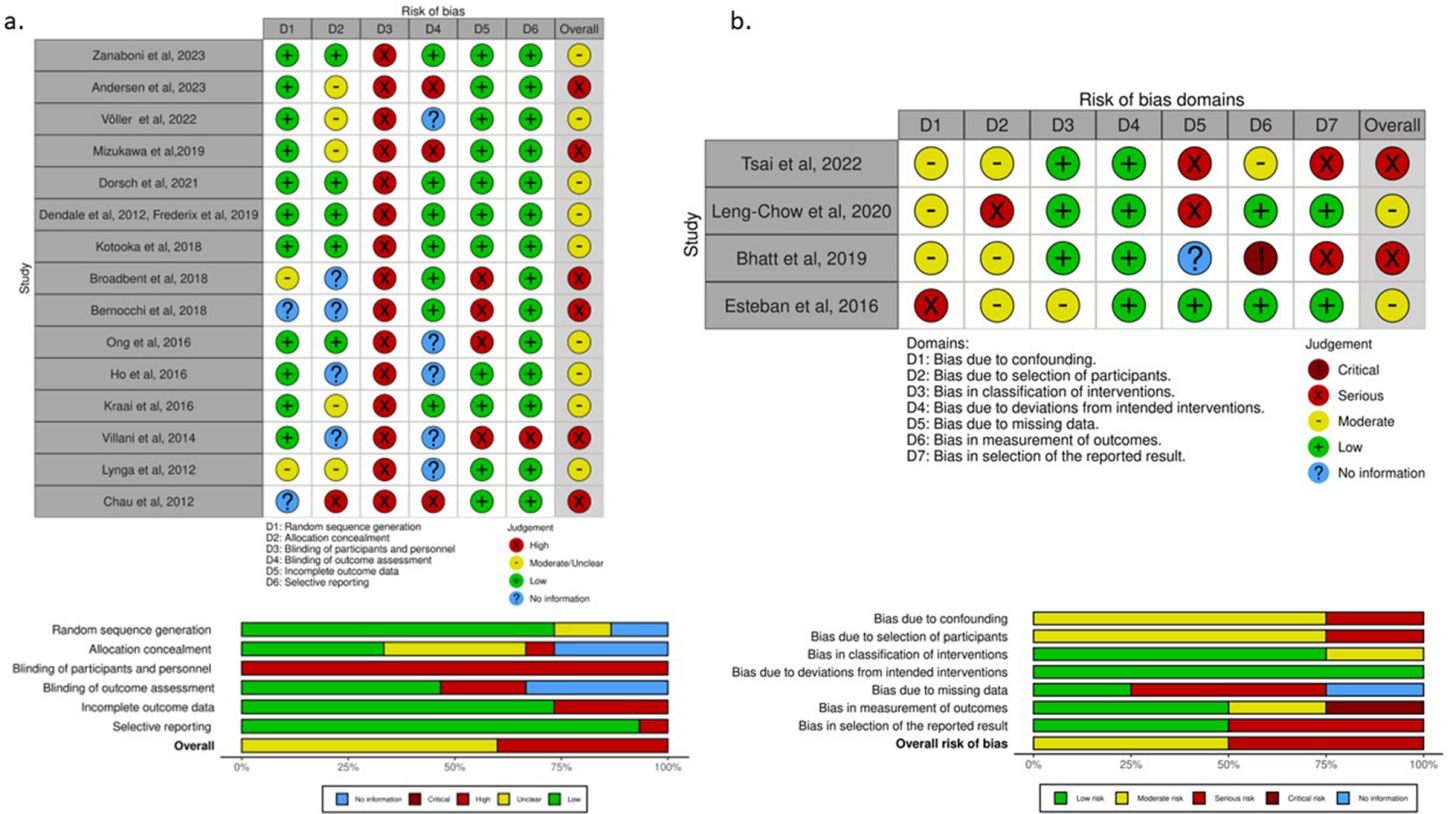
“Traffic light” plots of the domain-level judgements for each individual result and weighted bar plots of the distribution of risk-of-bias judgements within each bias domain for randomized controlled trials (RCTs) and non-RCTs. **(A)** For RCTs, the risk of bias tool for randomized trials (RoB2) was utilized. The majority of RCTs were generally categorized as having intermediate/unclear risk of bias. None of the studies was deemed as low risk due to the nature of telemedicine interventions, which precluded blinding of patients and healthcare professionals. **(B)** For non-RCTs, the risk of bias tool in non-randomized studies of interventions (ROBINS-I) was applied. Similar types of biases regarding the lack of blinding and pre-selection of the intervention population were encountered in all the studies. RCTs, randomized controlled trials; ROBINS-I, risk of bias tool in non-randomized studies of interventions; RoB2, risk of bias tool for randomized trials.

The risk of bias was assessed using the RoB2 tool for RCTs (Figure 4A), the ROBINS-I for non-RCTs (Figure 4B), the “NOS for cohort studies” for the cohort studies (Table 2), and “NOS for case-control studies” for the case-control study (Table 3).

**Table 2:** Newcastle-Ottawa scale (NOS) – for cohort studies.

Study	Item and score								Overall
	Representativeness of the exposed cohort (1)	Selection of the non-exposed cohort (1)	Ascertainment of exposure (1)	Demonstration that outcome of interest was not present at start of study (1)	Compare the ability of cohorts on the basis of the design or analysis (2)	Assessment of outcome (1)	Was follow-up long enough for outcomes to occur? (1)	Adequacy of follow-up of cohorts (1)	
Parkh et al., 2023	*	0	*	*	*	*	*	*	Good-quality 7/9
Naya Prieto et al., 2023	0	0	0	*	0	*	0	0	Poor-quality 2/9
Poelzl et al., 2022	*	*	0	*	*	*	*	*	Good-quality 7/9
Marcos et al., 2022	*	*	0	*	*	*	*	0	Good-quality 6/9
Ho et al., 2021	0	*	0	*	0	*	*	0	Fair-quality 4/9
Park et al., 2019	0	0	0	*	0	*	*	0	Poor-quality 3/9
Dyrvig et al., 2016	0	0	*	*	0	*	*	0	Fair-quality 4/9
Thomason et al.*,* 2015	0	0	*	*	0	0	*	*	Fair-quality 4/9
Davis et al.*,* 2015	0	0	*	*	0	*	*	0	Fair-quality 4/9

NOS, Newcastle-Ottawa Scale.

**Table 3:** Newcastle-Ottawa scale (NOS) – for case-control studies.

Study	Item and score								Overall
	Is the case definition adequate (1)	Representativeness of the cases (1)	Selection of Controls (1)	Definition of Controls (1)	Comparability of cases and controls on the basis of the design or analysis (2)	Assessment of exposure (1)	Same method of ascertainment of cases and controls (1)	Non-Response rate (1)	
Srivastava et al., 2019	*	0	0	*	*	*	*	0	Fair-quality 5/9 (usual care control)
	*	0	0	*	**	*	*	0	Fair-quality 6/9 (self-1-year before control)

NOS, Newcastle-Ottawa Scale.

Despite most studies being RCTs, the quality of their data was suboptimal with nine at moderate (43, 48, 50, 57–59, 62, 64, 65, 70) and six at serious risk of bias (45, 54, 60, 61, 69, 71). Some limitations associated with the use of telemedicine were common across all studies, particularly, the nature of the telemedicine intervention, which precluded blinding of participants and the treating teams.

Out of the four non-RCTs two were considered moderate risk (52, 63) and two serious risks of bias (46, 56). Non-RCTs experienced similar types of biases as RCTs and had additional risks due to the lack of randomization.

Furthermore, observational studies inherently pose a greater risk of bias, and the NOS score was assessed according to the guidelines of the “Agency for Healthcare Research and Quality”. Four of the cohort studies assessed with NOS were deemed as fair-quality (51, 66–68), three as good-quality (42, 47, 49), two as poor-quality (44, 53), one of the feasibility studies was assessed as fair-quality (51), the other one as poor-quality (53) and the case-control study assessed as fair-quality (55).

The overall confidence in the GRADE estimates for narrative data was moderate for readmissions in the HF population and low for readmissions in the COPD population (Table 4). Confidence assessment for the population with comorbid HF and COPD was not attempted as only one study focusing on this population was identified (61).

**Table 4:** Summary of findings table.

Outcome	Population	Effect	Number of Participants (Studies)	Methodological Limitations of the Studies	Indirectness	Imprecision	Inconsistency	Publication Bias	Certainty
Readmission burden assessed using a variety of different measures*	HF	Telemedicine incorporating telemonitoring has a neutral effect on readmissions in patients with HF	3,172 (18 studies)	Serious/moderate (assessed with RoB2, Robins-I, NOS)	Serious (heterogeneity in the telemedical modalities used, patient selection criteria and reporting of readmission-related outcomes)	Not serious	Not serious	Not strongly suspected	⊕⊕⊕○
Readmission burden assessed using a variety of different measures**	COPD	Telemedicine incorporating telemonitoring could reduce the readmission burden in patients with COPD	1,098 (11 studies)	Serious/moderate (assessed with RoB2, Robins-I, NOS)	Serious (heterogeneity in the telemedical modalities used, patient selection criteria and reporting of readmission-related outcomes)	Not serious	Moderate (The magnitude of the effect was comparable across studies reporting on the same outcome, no comparison can be made when different outcomes were reported)	Not serious/borderline (both negative and positive smaller trials were published)	⊕⊕○○

⊕⊕⊕○, Moderate certainty.

⊕⊕○○, Low certainty.

AECOPD, acute exacerbation of chronic obstructive pulmonary disease; COPD, chronic obstructive pulmonary disease; HF, heart failure; NOSNewcastle-Ottawa Scale; RCT, randomized controlled trial; RoB2risk of bias tool for randomized trials; ROBINS-I, risk of bias in non-randomized studies of interventions.

* HF-readmissions (30-, 90-, 365-day), all-cause readmissions (30-, 90-, 180-, 365-day), HF-readmission rate per 100 person-years, days alive neither in hospital nor inpatient care per potential days, all-cause 180-day hospital bed days, length of readmission, HF-readmission for >3 days, HF- hospital bed days, composite endpoints (e.g., all-cause readmissions or mortality).

** AECOPD-readmissions (180-, 730-day), all-cause readmissions (30, 90-, 180-day), respiratory-related readmissions, length of readmission/days spent in the hospital, time to first AECOPD-readmission, time to first readmission, composite endpoints (e.g., AECOPD-readmissions or mortality, time to AECOPD-readmission or mortality, all-cause readmission or ED visit).

## Discussion

With this systematic review, we aimed to evaluate the current evidence on the effectiveness of telemedicine incorporating telemonitoring technologies in reducing the readmission-related burden of patient populations with HF and/or COPD placing them at high risk for re-hospitalization. We observed a trend differentiating the effects of the implemented telemedicine among these groups. This variability could likely be attributed to differences in the nature or treatment of the diseases and their respective decompensations/exacerbations, or to the telemedicine modalities employed in the respective trials.

### Heart failure (HF)

ADHF is a syndrome characterized by a constellation of physical signs and subjective symptoms that can be easily monitored in the outpatient setting. Some of the physical signs include weight gain (due to peripheral and abdominal edema), hypoxemia, tachycardia, and tachypnea, and some of the symptoms can be fatigue, anorexia, and dyspnea (72). Our review suggests that telemedicine employing current telemonitoring modalities is unlikely to impact readmissions of patients with HF. The discrepancy between the expected effect and the results of this studies could potentially be attributed to various factors. First, one of the most common causes of readmission in patients with HF is medication non-adherence (73, 74). Individuals who are non-adherent to medication are less likely to accurately perform and report the daily self-monitoring and self-assessment required by telemedicine; thus, this might be a confounder. Additionally, the usual care for HF across many institutions in Europe and the US involves transitional care services, also known as “bridge clinics”, offering close post-discharge follow-up and additional disease-management education to the control groups. This approach has been shown to decrease the readmission risk in recently discharged HF patients (75, 76). Therefore, this close follow up in the control groups might decrease the significance of a readmission-related benefit observed in the telemedicine group.

Moreover, the treatment for ADHF almost always includes intravenous (IV) diuretics in the hospital setting to relieve the systemic congestion. Orally administered diuretics might not be as efficient, as the ones administered IV, for patients with ADHF, as these patients may have intestinal edema decreasing the absorption and bioavailability of orally administered agents (77, 78).

Furthermore, we noticed that the combination of telemonitoring with symptom questionnaires was less frequent in the HF studies (*n* = 3, 17%) (48, 52, 67) compared to the COPD studies (*n* = 6, 55%) (44, 49, 60, 63, 64, 67). Even though there has been skepticism regarding the practicality and reliability of self-reported information, this data could suggest that collection of patient-reported outcome measures though standardized questionnaires in conjunction with objective vital sign monitoring might be efficient in recognizing decompensation earlier. Notably, this could be applicable for ADHF as most patients recall onset of certain symptoms such as cough, edema, orthopnea or fatigue even seven days before the development of dyspnea which ultimately leads them to the ED (79, 80).

Previous systematic reviews examining the use of telemedicine in preventing hospitalization in the HF population have reported conflicting results. Regarding all-cause readmissions one meta-analysis provided low-quality data supporting that telemedicine can decrease all-cause readmissions (33). However, another study contradicted this result by showing a neutral effect on all-cause readmissions (34). This latter study did suggest, with moderate-quality data, that telemedicine could decrease HF-readmissions (34). Additionally, a different systematic review aligns with our results suggesting a neutral/unclear effect of telemedicine on readmissions (35). Our findings, combined with those of other studies, highlight the need for further high-quality research to draw definitive conclusions. Moreover, the implementation of novel telemonitoring technologies, as described in the “Emerging Vital Sign Monitoring Technologies” subsection, could enhance telemedicine's efficacy in preventing readmissions in this patient group.

### Chronic obstructive pulmonary disease (COPD)

AECOPD is characterized by hypoxemia, tachypnea, dyspnea, increase in cough frequency, and frequently tachycardia and fever (81). Our results suggest that readmissions of patients with COPD could potentially be reduced in patients remotely monitored with telemedicine devices. This effect, which was not apparent in the HF cohort, might be due to the nature of AECOPD management allowing for efficient outpatient treatment when identified early. In most cases except for inhaled agents, medications such as steroids and antibiotics (if infection is suspected) can be administered orally with comparable efficacy to IV delivery (82, 83).

Furthermore, the greater emphasis placed on the collection of subjective patient information, alongside vital sign monitoring, to early identify key symptoms of exacerbation such as increase in cough, sputum production or dyspnea, might have contributed to the COPD trials reaching their readmission-related endpoints. While the data regarding COPD and readmissions appear optimistic, it is important to consider that most of the studies included in the analysis had moderate or high risk of bias which may affect the reliability and generalizability of this conclusion.

A similar systematic review examining the COPD population contends that home management, mainly achieved through telemedicine, has the potential to decrease all-cause hospital readmissions (30). Moreover, a recent meta-analysis suggested that telemonitoring decreases all-cause AECOPD-readmissions but likely has a neutral effect on all-cause readmissions or hospital days (32).

Additionally, another systematic review, not focusing on the recently hospitalized patients with COPD or on telemonitoring, suggested with low certainty that there might be little or no effect on the number of patients admitted to the hospital (31). This finding aligns with a large retrospective study conducted in the Netherlands, which also did not specifically target the inpatient population and found no reduction in hospitalizations following the introduction of telemedicine in the COPD cohort (84). These findings, along with our study, underscore the importance of identifying a subgroup of COPD patients that would benefit the most from telemedicine and suggest that recently hospitalized patients appear to benefit more in terms of decrease in readmissions compared to the general COPD population. More studies providing high-quality evidence, possibly employing novel telemonitoring technologies, are necessary to validate and expand on these conclusions.

### Emerging remote vital sign monitoring technologies

While the identification of emerging telemonitoring technologies is beyond the scope of this review, it is briefly discussed because multiple novel telemonitoring instruments are reaching the point of clinical maturity. These advancements are anticipated to enable continuous monitoring of multiple biomarkers enhancing accuracy and patient compliance which could significantly impact the field of telemedicine. For instance, non-contact physiological measurements based on image sensors (e.g., digital cameras, smartphone cameras, radars) can facilitate continuous monitoring of physiological elements such as RR, breathing pattern, temperature, HR or even Ox Sat (85–88). Additionally, wearable photoplethysmography sensors will allow continuous tracking of multiple biomarkers (e.g., HR, RR) throughout the day, readily providing patient data at rest and during various levels of exertion (89). Furthermore, advancements in artificial intelligence (AI) will enable the development of integrative health data systems capable of detecting patterns indicative of potential cardiopulmonary decompensation as it occurs (90, 91). With the integration of these innovative technologies into future clinical trials, we may anticipate varying outcomes across different aspects of patient care, including readmissions.

## Limitations

This systematic review is subject to many of the common limitations and biases similar studies experience. Our selection criteria may have screened out studies that could potentially alter our results. Additional selection bias could be introduced as we focused on studies published only in the English language during 2012–2023. Furthermore, the lack of access to unpublished data could as well impose a publication bias on our study, as smaller studies not demonstrating readmission-related benefit in the telemedicine group are less likely to have been published. Unfortunately, the variability in the reported outcomes did not allow us to generate a funnel plot or do an Egger's test to determine the probability of publication bias, which decreases the certainty of our analyses. Regarding the quality of the studies analyzed, even though most were RCTs, none of them had a low risk of bias, due to innate limitations imposed by the telemedicine interventions (described in the “Risk of bias” section of the Materials). Additionally, in all RCTs and non-RCTs there was a deliberate selection of specific patient groups more likely to be capable of understanding and complying with the telemedicine interventions. Exclusion criteria typically involved patients with dementia, lack of access to the internet, psychiatric diseases, or residents in nursing homes. Moreover, different studies employed different inclusion and exclusion criteria for the HF and COPD population (e.g., NYHA class, number of previous readmissions, age). Therefore, the external validity and applicability of these studies to the general HF and COPD population is limited and this diversity in criteria complicate the comparison of outcomes among different studies. Our study is further limited by the inclusion of international studies; healthcare systems vary significantly, and there are substantial differences in how each country allocates resources, manages readmissions, or delivers outpatient care.

## Conclusions

In this study, we conducted a systematic literature review to examine the impact of telemedicine employing telemonitoring on hospital readmissions in high-risk patients with chronic diseases, particularly focusing on HF and COPD. To our knowledge, this is the first systematic review providing comparative data regarding the effectiveness of telemedicine in these two common groups of patients. Our conclusion, with moderate confidence, suggests that current telemonitoring modalities likely have a neutral effect on the readmissions of patients with HF, and with poor confidence, that telemedicine employing telemonitoring technologies could decrease the readmissions of patients with COPD. Nevertheless, even in the case of HF, most studies suggest that telemedicine could positively affect patients’ lives (e.g., mortality, QoL, ED visits). Thus, it remains to be determined how this broad spectrum of telemedicine interventions can be tailored to fit the needs of these patient populations. Additionally, understanding its effectiveness in patients with more than one chronic condition would be more representative of the actual patient populations being treated in the clinical setting. We anticipate that with the incorporation of novel telemonitoring technologies and the initiation of studies employing reproducible health systems approaches in telehealth services for at-risk populations (e.g., recently hospitalized patients), both of which have been prioritized due to the recent COVID-19 public health crisis, we will have stronger evidence regarding whether telemedicine has the potential to be a transformative intervention for the care of at-risk cardiopulmonary patients.

## Data Availability

The original contributions presented in the study are included in the article/Supplementary Material, further inquiries can be directed to the corresponding author.

## Supplementary material

Supplementary Figure S1 Column charts illustrating the proportion of studies achieving readmission-related endpoints differentiated for each vital sign being telemonitored. **(a)** Percentage of heart failure (HF) studies reaching their readmission-related endpoint (in red) compared to the percentages of studies telemonitoring for weight, heart rate (HR), blood pressure (BP), electrocardiograph (ECG), and oxygen saturation (Ox Sat). **(b)** Percentage of chronic obstructive pulmonary disease (COPD) studies reaching their readmission-related endpoint (in blue) compared to the percentages of studies telemonitoring for Ox Sat, HR, weight, pulmonary volumes via spirometry, BP, temperature, and respiratory rate (RR). The numerator on the fractions above each column indicates the number of studies telemonitoring for the specific vital sign with a positive readmission-related endpoint, the denominator indicates the total number of studies telemonitoring the same vital sign. BP, Blood Pressure; COPD, Chronic Obstructive Pulmonary Disease; ECG, Electrocardiograph; HF, Heart Failure; HR, Heart Rate; Ox Sat, Oxygen Saturation; RR, Respiratory Rate.

## References

[1] Sood S Mbarika V Jugoo S Dookhy R Doarn CR Prakash N What is telemedicine? A collection of 104 peer-reviewed perspectives and theoretical underpinnings. Telemed J E Health. (2007) 13(5):573–90. 10.1089/tmj.2006.0073 17999619

[2] Hincapie MA Gallego JC Gempeler A Pineros JA Nasner D Escobar MF. Implementation and usefulness of telemedicine during the COVID-19 pandemic: a scoping review. J Prim Care Community Health. (2020) 11:2150132720980612. 10.1177/2150132720980612 33300414 PMC7734546

[3] Lv M Wu T Jiang S Chen W Zhang J. Effects of telemedicine and mHealth on systolic blood pressure management in stroke patients: systematic review and meta-analysis of randomized controlled trials. JMIR Mhealth Uhealth. (2021) 9(6):e24116. 10.2196/24116 34114961 PMC8235282

[4] Angelucci A Aliverti A. Telemonitoring systems for respiratory patients: technological aspects. Pulmonology. (2020) 26(4):221–32. 10.1016/j.pulmoe.2019.11.006 31932232

[5] Barbosa MT Sousa CS Morais-Almeida M Simoes MJ Mendes P. Telemedicine in COPD: an overview by topics. COPD. (2020) 17(5):601–17. 10.1080/15412555.2020.1815182 32892650

[6] Hellem A Whitfield C Mansour M Curran Y Dinh M Warden K Determinants of bluetooth-enabled self-measured blood pressure monitoring in federally qualified health centers. J Prim Care Community Health. (2024) 15:21501319241229921. 10.1177/21501319241229921 38400549 PMC10894531

[7] Gerhardt G Yemane A Hickman P Oelschlaeger A Rollins E Brennan N. Medicare readmission rates showed meaningful decline in 2012. Medicare Medicaid Res Rev. (2013) 3(2):mmrr.003.02.b01. 10.5600/mmrr.003.02.b01 24753966 PMC3983725

[8] Mashhadi SF Hisam A Sikander S Rathore MA Rifaq F Khan SA Post discharge mHealth and teach-back communication effectiveness on hospital readmissions: a systematic review. Int J Environ Res Public Health. (2021) 18(19):10442. 10.3390/ijerph181910442 34639741 PMC8508113

[9] Mahmoudi S Taghipour HR Javadzadeh HR Ghane MR Goodarzi H Kalantar Motamedi MH. Hospital readmission through the emergency department. Trauma Mon. (2016) 21(2):e35139. 10.5812/traumamon.35139 27626018 PMC5003470

[10] Perera PN Armstrong EP Sherrill DL Skrepnek GH. Acute exacerbations of COPD in the United States: inpatient burden and predictors of costs and mortality. COPD. (2012) 9(2):131–41. 10.3109/15412555.2011.650239 22409371

[11] Giamouzis G Kalogeropoulos A Georgiopoulou V Laskar S Smith AL Dunbar S Hospitalization epidemic in patients with heart failure: risk factors, risk prediction, knowledge gaps, and future directions. J Card Fail. (2011) 17(1):54–75. 10.1016/j.cardfail.2010.08.010 21187265

[12] Heidenreich PA Fonarow GC Opsha Y Sandhu AT Sweitzer NK Warraich HJ Economic issues in heart failure in the United States. J Card Fail. (2022) 28(3):453–66. 10.1016/j.cardfail.2021.12.017 35085762 PMC9031347

[13] Braunwald E. The war against heart failure: the lancet lecture. Lancet. (2015) 385(9970):812–24. 10.1016/S0140-6736(14)61889-4 25467564

[14] Mosterd A Hoes AW. Clinical epidemiology of heart failure. Heart. (2007) 93(9):1137–46. 10.1136/hrt.2003.025270 17699180 PMC1955040

[15] Ambrosy AP Fonarow GC Butler J Chioncel O Greene SJ Vaduganathan M The global health and economic burden of hospitalizations for heart failure: lessons learned from hospitalized heart failure registries. J Am Coll Cardiol. (2014) 63(12):1123–33. 10.1016/j.jacc.2013.11.053 24491689

[16] Njoroge JN Teerlink JR. Pathophysiology and therapeutic approaches to acute decompensated heart failure. Circ Res. (2021) 128(10):1468–86. 10.1161/CIRCRESAHA.121.318186 33983837 PMC8126502

[17] Betz C. Filling the gaps in guideline-directed care. Am J Manag Care. (2021) 27(9 Suppl):S183–90. 10.37765/ajmc.2021.88672 34042416

[18] Retrum JH Boggs J Hersh A Wright L Main DS Magid DJ Patient-identified factors related to heart failure readmissions. Circ Cardiovasc Qual Outcomes. (2013) 6(2):171–7. 10.1161/CIRCOUTCOMES.112.967356 23386663 PMC4082819

[19] Gilotra NA Shpigel A Okwuosa IS Tamrat R Flowers D Russell SD. Patients commonly believe their heart failure hospitalizations are preventable and identify worsening heart failure, nonadherence, and a knowledge gap as reasons for admission. J Card Fail. (2017) 23(3):252–6. 10.1016/j.cardfail.2016.09.024 27742454

[20] Lin MH Yuan WL Huang TC Zhang HF Mai JT Wang JF. Clinical effectiveness of telemedicine for chronic heart failure: a systematic review and meta-analysis. J Investig Med. (2017) 65(5):899–911. 10.1136/jim-2016-000199 28330835

[21] Adeloye D Song P Zhu Y Campbell H Sheikh A Rudan I. Global, regional, and national prevalence of, and risk factors for, chronic obstructive pulmonary disease (COPD) in 2019: a systematic review and modelling analysis. Lancet Respir Med. (2022) 10(5):447–58. 10.1016/S2213-2600(21)00511-7 35279265 PMC9050565

[22] Osadnik CR Tee VS Carson-Chahhoud KV Picot J Wedzicha JA Smith BJ. Non-invasive ventilation for the management of acute hypercapnic respiratory failure due to exacerbation of chronic obstructive pulmonary disease. Cochrane Database Syst Rev. (2017) 7(7):Cd004104. 10.1002/14651858.CD004104.pub4 28702957 PMC6483555

[23] Skwarska E Cohen G Skwarski KM Lamb C Bushell D Parker S Randomized controlled trial of supported discharge in patients with exacerbations of chronic obstructive pulmonary disease. Thorax. (2000) 55(11):907–12. 10.1136/thorax.55.11.907 11050258 PMC1745644

[24] Donaldson GC Seemungal TA Bhowmik A Wedzicha JA. Relationship between exacerbation frequency and lung function decline in chronic obstructive pulmonary disease. Thorax. (2002) 57(10):847–52. 10.1136/thorax.57.10.847 12324669 PMC1746193

[25] Soler-Cataluña JJ Martínez-García MA Román Sánchez P Salcedo E Navarro M Ochando R. Severe acute exacerbations and mortality in patients with chronic obstructive pulmonary disease. Thorax. (2005) 60(11):925–31. 10.1136/thx.2005.040527 16055622 PMC1747235

[26] Lindenauer PK Dharmarajan K Qin L Lin Z Gershon AS Krumholz HM. Risk trajectories of readmission and death in the first year after hospitalization for chronic obstructive pulmonary disease. Am J Respir Crit Care Med. (2018) 197(8):1009–17. 10.1164/rccm.201709-1852OC 29206052 PMC5909167

[27] Wilkinson TM Donaldson GC Hurst JR Seemungal TA Wedzicha JA. Early therapy improves outcomes of exacerbations of chronic obstructive pulmonary disease. Am J Respir Crit Care Med. (2004) 169(12):1298–303. 10.1164/rccm.200310-1443OC 14990395

[28] Axson EL Ragutheeswaran K Sundaram V Bloom CI Bottle A Cowie MR Hospitalisation and mortality in patients with comorbid COPD and heart failure: a systematic review and meta-analysis. Respir Res. (2020) 21(1):54. 10.1186/s12931-020-1312-7 32059680 PMC7023777

[29] Khan SS Kalhan R. Comorbid chronic obstructive pulmonary disease and heart failure: shared risk factors and opportunities to improve outcomes. Ann Am Thorac Soc. (2022) 19(6):897–9. 10.1513/AnnalsATS.202202-152ED 35648080 PMC9169135

[30] Corcoran R Moore Z Avsar P Murray B. Home-based management on hospital re-admission rates in COPD patients: a systematic review. J Adv Nurs. (2024). 10.1111/jan.16168. [Epub ahead of print]. 38558439

[31] Janjua S Carter D Threapleton CJ Prigmore S Disler RT. Telehealth interventions: remote monitoring and consultations for people with chronic obstructive pulmonary disease (COPD). Cochrane Database Syst Rev. (2021) 7(7):Cd013196. 10.1002/14651858.CD013196.pub2 34693988 PMC8543678

[32] Lu JW Wang Y Sun Y Zhang Q Yan LM Wang YX Effectiveness of telemonitoring for reducing exacerbation occurrence in COPD patients with past exacerbation history: a systematic review and meta-analysis. Front Med (Lausanne). (2021) 8:720019. 10.3389/fmed.2021.720019 34568376 PMC8460761

[33] Liu S Li J Wan DY Li R Qu Z Hu Y Effectiveness of eHealth self-management interventions in patients with heart failure: systematic review and meta-analysis. J Med Internet Res. (2022) 24(9):e38697. 10.2196/38697 36155484 PMC9555330

[34] Aronow WS Shamliyan TA. Comparative effectiveness of disease management with information communication technology for preventing hospitalization and readmission in adults with chronic congestive heart failure. J Am Med Dir Assoc. (2018) 19(6):472–9. 10.1016/j.jamda.2018.03.012 29730178

[35] Morken IM Storm M Søreide JA Urstad KH Karlsen B Lunde Husebø AM. Posthospitalization follow-up of patients with heart failure using eHealth solutions: restricted systematic review. J Med Internet Res. (2022) 24(2):e32946. 10.2196/32946 35166680 PMC8889479

[36] Rezende LC Ribeiro EG Parreiras LC Guimarães RA Dos Reis GM Carajá AF Telehealth and telemedicine in the management of adult patients after hospitalization for COPD exacerbation: a scoping review. J Bras Pneumol. (2023) 9(3):e20220067. 10.36416/1806-3756/e20220067 37132694 PMC10171265

[37] Poberezhets V Kasteleyn MJ. Telemedicine and home monitoring for COPD - a narrative review of recent literature. Curr Opin Pulm Med. (2023) 29(4):259–69. 10.1097/MCP.0000000000000969 37140553

[38] da Costa Santos CM de Mattos Pimenta CA Nobre MR. The PICO strategy for the research question construction and evidence search. Rev Lat Am Enfermagem. (2007) 15(3):508–11. 10.1590/S0104-11692007000300023 17653438

[39] Sterne JAC Savović J Page MJ Elbers RG Blencowe NS Boutron I Rob 2: a revised tool for assessing risk of bias in randomised trials. Br Med J. (2019) 366:l4898. 10.1136/bmj.l4898 31462531

[40] Sterne JA Hernán MA Reeves BC Savović J Berkman ND Viswanathan M ROBINS-I: a tool for assessing risk of bias in non-randomised studies of interventions. Br Med J. (2016) 355:i4919. 10.1136/bmj.i4919 27733354 PMC5062054

[41] Murad MH Mustafa RA Schünemann HJ Sultan S Santesso N. Rating the certainty in evidence in the absence of a single estimate of effect. Evid Based Med. (2017) 22(3):85–7. 10.1136/ebmed-2017-110668 28320705 PMC5502230

[42] Parikh RV Axelrod AW Ambrosy AP Tan TC Bhatt AS Fitzpatrick JK Association between participation in a heart failure telemonitoring program and health care utilization and death within an integrated health care delivery system. J Card Fail. (2023) 29(12):1642–54. 10.1016/j.cardfail.2023.04.013 37220825

[43] Zanaboni P Dinesen B Hoaas H Wootton R Burge AT Philp R Long-term telerehabilitation or unsupervised training at home for patients with chronic obstructive pulmonary disease a randomized controlled trial. Am J Respir Crit Care Med. (2023) 207(7):865–75. 10.1164/rccm.202204-0643OC 36480957 PMC10111997

[44] Prieto AN Chang CL Fernández RA Gómez del Pulgar Murcia T Sánchez Mellado D Fernández Ormaechea MI Can a telemedicine program reduce the number of admissions in the second and third month after hospital discharge for an exacerbation of COPD compared to a conventional follow-up system? Open Respiratory Archives. (2023) 5(1):100222. 10.1016/j.opresp.2022.100222 37497246 PMC10369518

[45] Andersen FD Trolle C Pedersen AR Køpfli ML Børgesen S Jensen MS Effect of telemonitoring on readmissions for acute exacerbation of chronic obstructive pulmonary disease: a randomized clinical trial. J Telemed Telecare. (2023) 10.1177/1357633X221150279. [Epub ahead of print]. 36683440

[46] Tsai WJ Wen YK Cheng YY Huang JL Chen YW. Effectiveness of home-based telerehabilitation programs on functional capacity and cardiac function in elderly heart failure patients: a prospective longitudinal study. Medicine (Baltimore). (2022) 101(28):e29799. 10.1097/MD.0000000000029799 35838996 PMC11132345

[47] Poelzl G Egelseer-Bruendl T Pfeifer B Modre-Osprian R Welte S Fetz B Feasibility and effectiveness of a multidimensional post-discharge disease management programme for heart failure patients in clinical practice: the HerzMobil tirol programme. Clin Res Cardiol. (2022) 111(3):294–307. 10.1007/s00392-021-01912-0 34269863

[48] Völler H Bindl D Nagels K Hofmann R Vettorazzi E Wegscheider K The first year of noninvasive remote telemonitoring in chronic heart failure is not cost saving but improves quality of life: the randomized controlled CardioBBEAT trial. Telemed J E Health. (2022) 28(11):1613–22. 10.1089/tmj.2022.0021 35325562 PMC9700331

[49] Marcos PJ Represas Represas C Ramos C Cimadevila Álvarez B Fernández Villar A Fraga Liste A Impact of a home telehealth program after a hospitalized COPD exacerbation: a propensity score analysis. Arch Bronconeumol. (2022) 58(6):474–81. 10.1016/j.arbres.2020.05.030 32600850

[50] Dorsch MP Farris KB Rowell BE Hummel SL Koelling TM. The effects of the ManageHF4Life Mobile app on patients with chronic heart failure: randomized controlled trial. JMIR Mhealth Uhealth. (2021) 9(12):e26185. 10.2196/26185 34878990 PMC8693200

[51] Ho K Novak Lauscher H Cordeiro J Hawkins N Scheuermeyer F Mitton C Testing the feasibility of sensor-based home health monitoring (TEC4Home) to support the convalescence of patients with heart failure: pre-post study. JMIR Form Res. (2021) 5(6):e24509. 10.2196/24509 34081015 PMC8212633

[52] Chow WL Aung CYK Tong SC Goh GS Lee S MacDonald MR Effectiveness of telemonitoring-enhanced support over structured telephone support in reducing heart failure-related healthcare utilization in a multi-ethnic Asian setting. J Telemed Telecare. (2020) 26(6):332–40. 10.1177/1357633X18825164 30782070

[53] Park C Otobo E Ullman J Rogers J Fasihuddin F Garg S Impact on readmission reduction among heart failure patients using digital health monitoring: feasibility and adoptability study. JMIR Med Inform. (2019) 7(4):e13353. 10.2196/13353 31730039 PMC6913758

[54] Mizukawa M Moriyama M Yamamoto H Rahman MM Naka M Kitagawa T Nurse-led collaborative management using telemonitoring improves quality of life and prevention of rehospitalization in patients with heart failure. Int Heart J. (2019) 60(6):1293–302. 10.1536/ihj.19-313 31735786

[55] Srivastava A Do JM Sales VL Ly S Joseph J. Impact of patient-centred home telehealth programme on outcomes in heart failure. J Telemed Telecare. (2019) 25(7):425–30. 10.1177/1357633X18775852 29793388

[56] Bhatt SP Patel SB Anderson EM Baugh D Givens T Schumann C Video telehealth pulmonary rehabilitation intervention in chronic obstructive pulmonary disease reduces 30-day readmissions. Am J Respir Crit Care Med. (2019) 200(4):511–3. 10.1164/rccm.201902-0314LE 30978302 PMC6701038

[57] Dendale P De Keulenaer G Troisfontaines P Weytjens C Mullens W Elegeert I Effect of a telemonitoring-facilitated collaboration between general practitioner and heart failure clinic on mortality and rehospitalization rates in severe heart failure: the TEMA-HF 1 (TElemonitoring in the MAnagement of heart failure) study. Eur J Heart Fail. (2012) 14(3):333–40. 10.1093/eurjhf/hfr144 22045925

[58] Frederix I Vanderlinden L Verboven AS Welten M Wouters D De Keulenaer G Long-term impact of a six-month telemedical care programme on mortality, heart failure readmissions and healthcare costs in patients with chronic heart failure. J Telemed Telecare. (2019) 25(5):286–93. 10.1177/1357633X18774632 29742959

[59] Kotooka N Kitakaze M Nagashima K Asaka M Kinugasa Y Nochioka K The first multicenter, randomized, controlled trial of home telemonitoring for Japanese patients with heart failure: home telemonitoring study for patients with heart failure (HOMES-HF). Heart Vessels. (2018) 33(8):866–76. 10.1007/s00380-018-1133-5 29450689

[60] Broadbent E Garrett J Jepsen N Li Ogilvie V Ahn HS Robinson H Using robots at home to support patients with chronic obstructive pulmonary disease: pilot randomized controlled trial. J Med Internet Res. (2018) 20(2):e45. 10.2196/jmir.8640 29439942 PMC5829456

[61] Bernocchi P Vitacca M La Rovere MT Volterrani M Galli T Baratti D Home-based telerehabilitation in older patients with chronic obstructive pulmonary disease and heart failure: a randomised controlled trial. Age Ageing. (2018) 47(1):82–8. 10.1093/ageing/afx146 28985325

[62] Ong MK Romano PS Edgington S Aronow HU Auerbach AD Black JT Effectiveness of remote patient monitoring after discharge of hospitalized patients with heart failure: the better effectiveness after transition – heart failure (BEAT-HF) randomized clinical trial. JAMA Intern Med. (2016) 176(3):310–8. 10.1001/jamainternmed.2015.7712 26857383 PMC4827701

[63] Esteban C Moraza J Iriberri M Aguirre U Goiria B Quintana JM Outcomes of a telemonitoring-based program (telEPOC) in frequently hospitalized COPD patients. Int J Chron Obstruct Pulmon Dis. (2016) 11:2919–30. 10.2147/COPD.S115350 27920519 PMC5125987

[64] Ho TW Huang CT Chiu HC Ruan SY Tsai YJ Yu CJ Effectiveness of telemonitoring in patients with chronic obstructive pulmonary disease in Taiwan-a randomized controlled trial. Sci Rep. (2016) 6:23797. 10.1038/srep23797 27029815 PMC4814821

[65] Kraai I de Vries A Vermeulen K van Deursen V van der Wal M de Jong R The value of telemonitoring and ICT-guided disease management in heart failure: results from the IN TOUCH study. Int J Med Inform. (2016) 85(1):53–60. 10.1016/j.ijmedinf.2015.10.001 26514079

[66] Dyrvig AK Gerke O Kidholm K Vondeling H. A cohort study following up on a randomised controlled trial of a telemedicine application in COPD patients. J Telemed Telecare. (2015) 21(7):377–84. 10.1177/1357633X15572202 25761469

[67] Davis C Bender M Smith T Broad J. Feasibility and acute care utilization outcomes of a post-acute transitional telemonitoring program for underserved chronic disease patients. Telemed J E Health. (2015) 21(9):705–13. 10.1089/tmj.2014.0181 25955129

[68] Thomason TR HS Perkins KE Hamilton E Nelson B. Home telehealth and hospital readmissions: a retrospective OASIS-C data analysis. Home Healthc now. (2015) 33(1):20–6. 10.1097/NHH.0000000000000167 25654342

[69] Villani A Malfatto G Compare A Della Rosa F Bellardita L Branzi G Clinical and psychological telemonitoring and telecare of high risk heart failure patients. J Telemed Telecare. (2014) 20(8):468–75. 10.1177/1357633X14555644 25339632

[70] Lynga P Persson H Hagg-Martinell A Hagglund E Hagerman I Langius-Eklof A Weight monitoring in patients with severe heart failure (WISH). A randomized controlled trial. Eur J Heart Fail. (2012) 14(4):438–44. 10.1093/eurjhf/hfs023 22371525

[71] Chau JP Lee DT Yu DS Chow AY Yu WC Chair SY A feasibility study to investigate the acceptability and potential effectiveness of a telecare service for older people with chronic obstructive pulmonary disease. Int J Med Inform. (2012) 81(10):674–82. 10.1016/j.ijmedinf.2012.06.003 22789911

[72] Mant J Doust J Roalfe A Barton P Cowie MR Glasziou P Systematic review and individual patient data meta-analysis of diagnosis of heart failure, with modelling of implications of different diagnostic strategies in primary care. Health Technol Assess. (2009) 13(32):1–207, iii. 10.3310/hta13320 19586584

[73] Annema C Luttik M-L Jaarsma T. Reasons for readmission in heart failure: perspectives of patients, caregivers, cardiologists, and heart failure nurses. Heart Lung. (2009) 38(5):427–34. 10.1016/j.hrtlng.2008.12.002 19755193

[74] Wu JR Moser DK. Medication adherence mediates the relationship between heart failure symptoms and cardiac event-free survival in patients with heart failure. J Cardiovasc Nurs. (2018) 33(1):40–6. 10.1097/JCN.0000000000000427 28591004 PMC5714687

[75] Feltner C Jones CD Cené CW Zheng ZJ Sueta CA Coker-Schwimmer EJ Transitional care interventions to prevent readmissions for persons with heart failure: a systematic review and meta-analysis. Ann Intern Med. (2014) 160(11):774–84. 10.7326/M14-0083 24862840

[76] Van Spall HGC Rahman T Mytton O Ramasundarahettige C Ibrahim Q Kabali C Comparative effectiveness of transitional care services in patients discharged from the hospital with heart failure: a systematic review and network meta-analysis. Eur J Heart Fail. (2017) 19(11):1427–43. 10.1002/ejhf.765 28233442

[77] Ikeda Y Ishii S Maemura K Oki T Yazaki M Fujita T Association between intestinal oedema and oral loop diuretic resistance in hospitalized patients with acute heart failure. ESC Heart Fail. (2021) 8(5):4067–76. 10.1002/ehf2.13525 34323025 PMC8497223

[78] Suri SS Pamboukian SV. Optimal diuretic strategies in heart failure. Ann Transl Med. (2020) 9(6):517. 10.21037/atm-20-4600 PMC8039650 33850914

[79] Born MC Azzolin KDO de Souza EN. How long before hospital admission do the symptoms of heart failure decompensation arise? Rev Lat Am Enfermagem. (2019) 27:e3119. 10.1590/1518-8345.2735.3119

[80] Schiff GD Fung S Speroff T McNutt RA. Decompensated heart failure: symptoms, patterns of onset, and contributing factors. Am J Med. (2003) 114(8):625–30. 10.1016/S0002-9343(03)00132-3 12798449

[81] Celli BR Fabbri LM Aaron SD Agusti A Brook R Criner GJ An updated definition and severity classification of chronic obstructive pulmonary disease exacerbations: the Rome proposal. Am J Respir Crit Care Med. (2021) 204(11):1251–8. 10.1164/rccm.202108-1819PP 34570991

[82] de Jong YP Uil SM Grotjohan HP Postma DS Kerstjens HA van den Berg JW. Oral or IV prednisolone in the treatment of COPD exacerbations: a randomized, controlled, double-blind study. Chest. (2007) 132(6):1741–7. 10.1378/chest.07-0208 17646228

[83] Fu Y Chapman EJ Boland AC Bennett MI. Evidence-based management approaches for patients with severe chronic obstructive pulmonary disease (COPD): a practice review. Palliat Med. (2022) 36(5):770–82. 10.1177/02692163221079697 35311415 PMC9087316

[84] van der Burg JMM Aziz NA Kaptein MC Breteler MJM Janssen JH van Vliet L Long-term effects of telemonitoring on healthcare usage in patients with heart failure or COPD. Clinical EHealth. (2020) 3:40–8. 10.1016/j.ceh.2020.05.001

[85] Takano C Ohta Y. Heart rate measurement based on a time-lapse image. Med Eng Phys. (2007) 29(8):853–7. 10.1016/j.medengphy.2006.09.006 17074525

[86] Van Gastel M Stuijk S De Haan G. Camera-based pulse-oximetry - validated risks and opportunities from theoretical analysis. Biomed Opt Express. (2018) 9(1):102–19. 10.1364/BOE.9.000102 29359090 PMC5772567

[87] Manullang MCT Lin YH Lai SJ Chou NK. Implementation of thermal camera for non-contact physiological measurement: a systematic review. Sensors (Basel). (2021) 21(23):7777. 10.3390/s21237777 34883780 PMC8659982

[88] Choo YJ Lee GW Moon JS Chang MC. Application of non-contact sensors for health monitoring in hospitals: a narrative review. Front Med (Lausanne). (2024) 11:1421901. 10.3389/fmed.2024.1421901 38933102 PMC11199382

[89] Charlton PH Allen J Bailón R Baker S Behar JA Chen F The 2023 wearable photoplethysmography roadmap. Physiol Meas. (2023) 44(11):111001. 10.1088/1361-6579/acead2 37494945 PMC10686289

[90] Chaichulee S Villarroel M Jorge J Arteta C McCormick K Zisserman A Cardio-respiratory signal extraction from video camera data for continuous non-contact vital sign monitoring using deep learning. Physiol Meas. (2019) 40(11):115001. 10.1088/1361-6579/ab525c 31661680 PMC7655150

[91] S M RG Mathivanan SK Shivahare BD Chandan RR Shah MA. A comprehensive health assessment approach using ensemble deep learning model for remote patient monitoring with IoT. Sci Rep. (2024) 14(1):15661. 10.1038/s41598-024-66427-w 38977848 PMC11231150

[92] McGuinness LA Higgins JPT. Risk-of-bias VISualization (robvis): an R package and shiny web app for visualizing risk-of-bias assessments. Res Synth Methods. (2021) 12(1):55–61. 10.1002/jrsm.1411 32336025

