# Supplementary figures and images for: The effect of telemedicine employing telemonitoring instruments on readmissions of patients with heart failure and/or COPD: a systematic review
Source: Front Digit Health. 2024 Sep 25;6:1441334. doi: 10.3389/fdgth.2024.1441334 (PMC11461467; doi:10.3389/fdgth.2024.1441334)

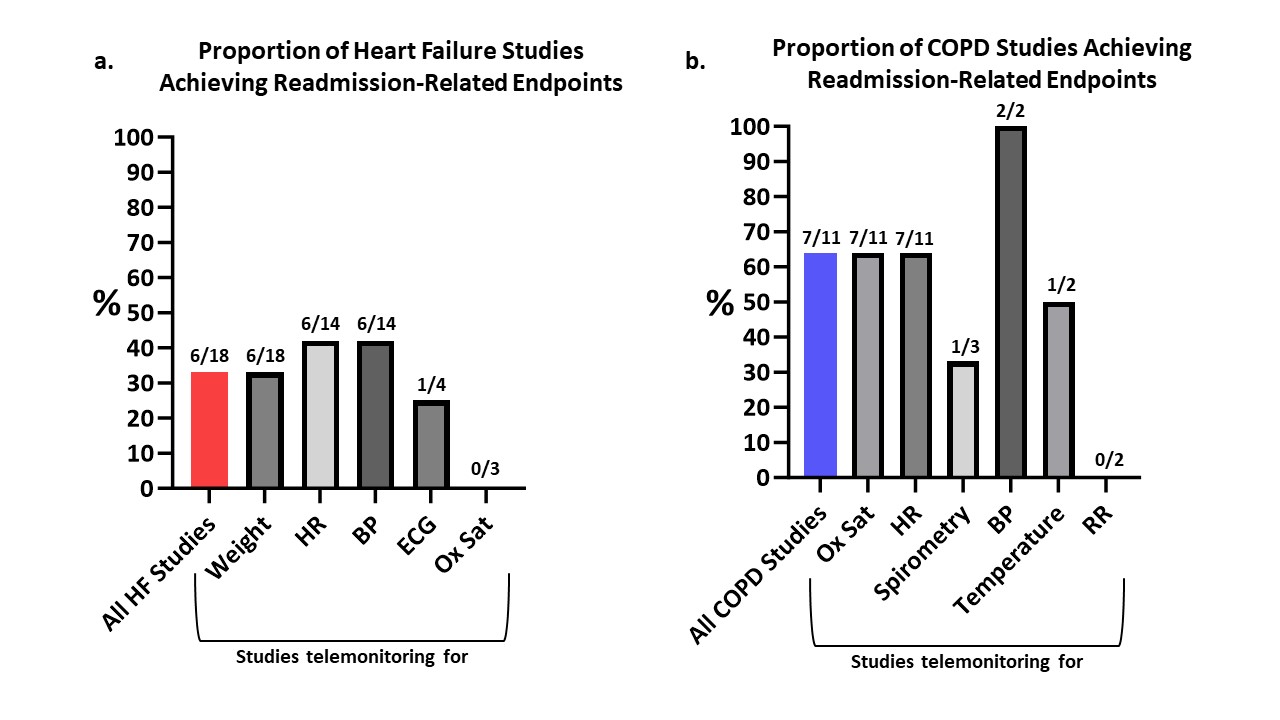

Supplement: Supplementary Figure S1 [file Image1.jpeg]
