# Supplementary material for: The effect of telemedicine employing telemonitoring instruments on readmissions of patients with heart failure and/or COPD: a systematic review
Source: Front Digit Health. 2024 Sep 25;6:1441334. doi: 10.3389/fdgth.2024.1441334 (PMC11461467; doi:10.3389/fdgth.2024.1441334)
Supplement: Supplementary file 2 [file Datasheet1.docx]

Pubmed

(("Patient Readmission"[MeSH Terms] OR ("Patient Readmission"[Text Word] OR "patient readmissions"[Text Word] OR "patient re-admission"[Text Word] OR "readmission"[Text Word] OR "readmissions"[Text Word] OR "re-admission"[Text Word] OR "re-admissions"[Text Word])) AND ("Telemedicine"[MeSH Terms] OR "Mobile Applications"[MeSH Terms] OR "Smartphone"[MeSH Terms] OR ("Telemedicine"[Text Word] OR "Mobile Applications"[Text Word] OR "mobile application"[Text Word] OR "Smartphone"[Text Word] OR "smartphones"[Text Word] OR "mobile app"[Text Word] OR "mobile apps"[Text Word] OR "portable electronic apps"[Text Word] OR "smart phone apps"[Text Word] OR "smart phone app"[Text Word] OR "smartphone app"[Text Word] OR "smart phone apps"[Text Word] OR "digital health"[Text Word] OR "ehealth"[Text Word] OR "mhealth"[Text Word] OR "health applications"[Text Word] OR "health apps"[Text Word] OR "health app"[Text Word]))) AND ((alladult[Filter]) AND (2012:2023[pdat]))

Scopus (all textwords)

( {hospital readmission} OR {patient readmission} ) AND ( telemedicine OR smartphone OR {telehealth} OR {digital health} OR {ehealth} OR {health application} OR {mhealth} OR {health app} OR {mobile application} OR {mobile app} OR {portable electronic app} OR {smartphone app} OR smartphone )

Limit to English and 2012-2023

ABI Inform (business database-thesaurus and textwords)

("hospital readmissions" OR "hospital readmission" OR "patient readmission" OR "patient readmissions") AND ((MAINSUBJECT.EXACT("Telemedicine") OR MAINSUBJECT.EXACT("Smartphones") OR “telehealth” OR "digital health" OR “eHealth” OR "health applications" OR “mHealth” OR "health apps" OR "health app" OR "mobile applications") OR ("mobile application" OR "mobile app" OR "mobile apps" OR "portable electronic app" OR "smartphone apps"))

Limit to 2012-2023

Excluded: wire feeds; blogs; other; newspaper; magazines; dissertations; working papers
